# Can consumer wearable activity tracker-based interventions improve physical activity and cardiometabolic health in patients with chronic diseases? A systematic review and meta-analysis of randomised controlled trials

**DOI:** 10.1186/s12966-020-00955-2

**Published:** 2020-05-11

**Authors:** Wouter M. A. Franssen, Gregor H. L. M. Franssen, Jan Spaas, Francesca Solmi, Bert O. Eijnde

**Affiliations:** 1 REVAL – Rehabilitation Research Center, Faculty of Rehabilitation Sciences, Hasselt University, Hasselt, Belgium; 2 BIOMED – Biomedical Research Institute, Faculty of Medicine and Life Sciences, Hasselt University, Hasselt, Belgium; 3 Department of Education and Research Support, University Library, Maastricht University, Maastricht, The Netherlands; 4 Data Science Institute for Biostatistics and Statistical Bioinformatics, Hasselt University, Hasselt, Belgium; 5 ADLON Sports Medical Center, Hasselt, Belgium

**Keywords:** Activity tracker, Physical activity, Cardiometabolic health, Chronic disease

## Abstract

**Background:**

To date, it is unclear if consumer wearable activity trackers (CWATs), with or without behaviour multi-component strategies, effectively improve adherence to physical activity and health outcomes under free living conditions in populations with chronic diseases. Therefore, we systematically evaluated the efficacy of CWAT-based interventions to promote physical activity levels and cardiometabolic health in populations with chronic diseases.

**Methods:**

Randomised controlled trials were collected from five bibliographic databases (PubMed, Embase, Web of Science, The Cochrane Central Register of Controlled Trials and CINAHL). Studies were eligible for inclusion if they evaluated a CWAT-based counselling intervention versus control intervention among patients with chronic respiratory diseases, type 2 diabetes mellitus, cardiovascular diseases, overweight/obesity, cognitive disorders, or sedentary older adults. Data were pooled using a random-effects model.

**Results:**

After deduplication 8147 were identified of which 35 studies met inclusion criteria (chronic respiratory diseases: 7, type 2 diabetes mellitus: 12, cardiovascular diseases: 6, overweight/obesity: 3, cognitive disorders: 1, sedentary older adults: 6). Compared to control groups, CWAT-based interventions significantly increased physical activity by 2123 steps per day (95% confidence interval [CI], [1605–2641]; *p* < 0.001). In addition, CWAT-based interventions in these populations significantly decreased systolic blood pressure (− 3.79 mm Hg; 95% CI: [− 4.53, − 3.04] mm Hg; *p* < 0.001), waist circumference (− 0.99 cm; 95% CI: [− 1.48, − 0.50] cm; p < 0.001) and low-density lipoprotein cholesterol concentration (− 5.70 mg/dl; 95% CI: [− 9.24, − 2.15] mg/dl; *p* = 0.002).

**Conclusion:**

CWAT-based interventions increase physical activity and have beneficial effects on important health-related outcomes such as systolic blood pressure, waist circumference and LDL cholesterol concentration in patients with chronic diseases.

## Summary box

**What is already known?**

- Physical inactivity is one of the major contributing factors for the development of chronic diseases and highly correlated with mortality and hospitalization
- Consumer wearable activity trackers have predominantly been applied in the sports community for self-monitoring sport/training performance-related parameters

**What are the new findings?**

- Consumer wearable activity tracker-based interventions increase physical activity
- The increased number of steps could result in an improvement of the general health and a reduction of all-cause mortality in people with chronic diseases

## Introduction

Chronic diseases such as chronic respiratory diseases, type 2 diabetes mellitus (T2DM), cardiovascular diseases (CVD) and cognitive disorders are an important public health concern worldwide [1]. In fact, recent data indicate that the prevalence of chronic diseases has increased considerably to 40 million global deaths in 2015. As a result, chronic diseases now are the leading cause of mortality worldwide and as such constitute one of the most important challenges for the twenty-first century [2, 3].

Physical inactivity is one of the major contributing factors for the development of chronic diseases and is highly correlated with mortality and hospitalization [4, 5]. On the other hand, it is well known that increased physical activity has significant health benefits and is associated with the prevention and delayed onset of many chronic diseases [4, 6]. Given the important role of physical activity in the prevention and management of chronic diseases, it is crucial to promote physical activity. Hence, to date, a multitude of physical activity recommendations and many supervised training interventions and rehabilitation programs are available to encourage physical activity in the global population [7, 8]. Nevertheless, a recent report from the World Health Organization (WHO) indicates that 23% of the adult and 80% of the adolescent population is still physically inactive [9]. Here, very poor long-term compliance to adequate physical activity and a healthy lifestyle appears to be one of the main factors explaining this discrepancy. Consequently, any strategy that improves long-term adherence to adequate daily physical activity and a healthy lifestyle, especially in a population with chronic diseases, is worthwhile investigating. The use of structured behaviour change interventions (e.g. telephone counselling, group sessions, provision of written information materials or individual education sessions) is reported to be effective in increasing physical activity, subsequently leading to a reduced progression of chronic diseases [10–13]. In addition, although Bravata et al. showed that goal setting may be a key motivational factor for increasing physical activity, this conclusion was based on observational studies and healthy individuals [14]. However, these strategies are often resource-intensive and time-consuming, factors that limit long-term adherence and usually not feasible in routine clinical care [15]. In this respect and following the recent use of pedometer and accelerometer-based remote monitoring of physical activity in patients with chronic diseases, consumer wearable activity trackers (CWATs) may be an alternative strategy to increase physical activity levels. CWATs are electronic devices used for monitoring and recording daily physical activity, although nowadays the term is also used for wearable fitness gadgets. These CWATs are consisted of pedometers (e.g. Omron and Yamax), which provide direct feedback on the level of physical activity in terms of number of steps per day, and activity trackers (Polar, Fitbit, Garmin and Apple Watch) which often include goal setting and can monitor physical activity and fitness related metrics including the amount and intensity of physical activity, sedentary behaviour and heart rate. Initially, CWATs have predominantly been applied in the sports community for self-monitoring sport/training performance-related parameters [16–18]. To date, they are widely used to quantify physical activity and monitor fitness. Possibly, the self-management, motivational and goal setting properties of these commercially available devices may also help patients with chronic diseases to better adhere to long-term physical activity under free-living conditions in a home-based setting. Surprisingly and despite the widespread use of these wearables, their feasibility and efficacy on physical activity (compliance) and cardiometabolic health including anthropometric measurements, systemic blood pressure, lipid profile and glycemic index, especially in patients with chronic diseases, is not fully clear. Although recent reviews have shown that CWATs have potential to increase physical activity, they included only one type of chronic disease [19, 20] or solely pedometers [14, 21]. In addition, conclusions are not based on randomised controlled trials [14, 22] and most of reviews did not (or partly) report a cardiometabolic risk profile [15, 19, 20, 23].

Therefore, this study aims to systematically evaluate the efficacy of CWAT-based interventions, as CWATs being either the primary components of an intervention or as part of a multi-component intervention, to promote physical activity levels and cardiometabolic health in populations with chronic diseases including chronic respiratory diseases, T2DM, CVD, overweight/obesity, cognitive disorders or sedentary older adults. A better understanding whether CWATs improve adherence to physical activity and hereby affect cardiometabolic health outcomes may help to increase the efficacy and quality of health care in populations with chronic diseases.

## Methods

This systematic review, including an explorative meta-analysis, was registered in the PROSPERO international prospective register of systematic reviews (registration number: CRD42019124126) and was performed in accordance with The Preferred Reporting Items for Systematic reviews and Meta-Analysis (PRISMA) statement [24, 25].

### Data sources and search strategies

Studies were collected (from inception until March 2019) using computer-based searches in the PubMed, Embase, Web of Science, The Cochrane Central Register of Controlled Trials (CENTRAL) and CINAHL electronic databases. Database specific search strategies were developed with the guidance of a professional clinical librarian. The database searches were performed using four main concepts: chronic diseases or sedentary older adults, CWATs, behaviour change and cardiometabolic health measures. For each main concept relevant related terms and keywords were included in the sensitive search (details presented in Appendix I). The systematic search was limited to the English, German and Dutch language.

### Eligibility criteria

Inclusion criteria to select studies were: 1) Study population: adult (aged 18 or older) patients with main chronic diseases including chronic respiratory diseases, T2DM, CVD, overweight/obesity and cognitive disorders, or sedentary older adults (> 55 years; high risk population); 2) Types of studies: peer-reviewed randomised controlled trials regarding a CWAT-based behaviour change intervention compared to a control intervention or usual care comparison group. The behaviour change intervention could be a CWAT-only intervention or a multi-component intervention consisting of a CWAT in combination with lifestyle data platforms, applications to change lifestyle behaviour or coaching sessions. In addition, physical activity should be measured objectively; 3) Primary outcome: physical activity expressed in number of steps per day and 4) Secondary outcomes: cardiometabolic health outcomes including physical fitness, exercise capacity, anthropometric measures (body weight, body mass index (BMI), waist circumference and percentage fat mass), systolic and diastolic blood pressure (BP), resting heart rate, lipid profile (blood total cholesterol, high-density lipoprotein (HDL) cholesterol, low-density lipoprotein (LDL) cholesterol and triglyceride concentrations), blood glycated haemoglobin concentration (HbA1c) and the fasting glucose and insulin concentration. Studies that included dietary interventions or had an intervention duration of less than 6 weeks were excluded.

### Study selection

Studies were independently screened in three different steps by two authors (W.M.A.F. and J.S.). Firstly, duplicates were removed using the de-duplication method from Bramer et al. [26] and a first selection was performed based on titles and abstracts to identify relevant studies. Then, articles were screened and systematically excluded when they did not meet the pre-specified inclusion criteria. In addition, reviews, editorials, congress abstracts and validation studies were also excluded. Disagreements between authors were resolved by consensus with a third reviewer (B.O.E).

### Data extraction

Data were independently extracted by two of the reviewers (W.M.A.F. and J.S.). Data extraction was performed with the aid of a predesigned data collection form, adapted from the extraction form of the Cochrane Collaboration (Appendix II). For each study, the reviewers extracted information with respect to study characteristics (type of study, population description, focused disease or condition, types of outcome measures); study participants (sample size, demographics); methods (study aim, study design, intervention duration, type and frequency, CWAT type, blinding, amount of intervention groups, number of included participants, dropouts and the number of individuals that were randomised and analyzed); outcome variables (outcome definition, unit of measurement, time points measured and reported, statistical method used). If a study consisted of two or more study arms of which one of the intervention arms did not meet the inclusion criteria, data were only extracted from the study arms that met the inclusion criteria. Continuous data including, means, standard deviations and the sample size numbers were extracted. When mean differences were not available, authors of the included studies were contacted to request additional data. Without availability of standard deviations, measures of variance were estimated from the standard error of a mean, confidence intervals or *p*-values according to the Cochrane Handbook for Systematic Reviews of Interventions the Cochrane Collaboration (Version 5.2, chapter 7) [27]. In addition, when data were presented as median and interquartile range, the mean and standard deviation were estimated using the formula from Hozo et al. [28]. Blood parameters were converted to the same unit (from mmol/l to mg/dl), including triglycerides (divide by 0.0112), total cholesterol, HDL cholesterol, LDL cholesterol (divide by 0.02586) and glucose (divide by 0.05551) concentration [29].

### Study quality assessment

The risk of bias of the included studies was assessed by one reviewer (W.M.A.F.) as recommended by Higgins et al. (Cochrane ‘Risk of Bias’ assessment tool, the Cochrane Collaboration). Here, the following methodological criteria were assessed: sequence generation, allocation concealment, blinding of participants, personnel and outcome assessors, incomplete outcome data, selective outcome reporting and other potential threats to validity [27]. Each of these criteria were judged and classified as ‘low risk’, ‘high risk’ or ‘unclear risk’ of bias.

### Statistical analysis

Statistical analyses were performed using R version 3.6.0 (The R foundation for Statistical Computing, Wien, Austria) [30]. The mean differences (post-intervention parameter – pre-intervention parameter) with 95% confidence intervals were calculated and pooled effect estimates were obtained using a random-effects model due to the large heterogeneity among the studies (different populations, age, intervention type). Some studies used multiple intervention groups, or the same intervention and control groups were measured at multiple time intervals. Therefore, an extension of the standard meta-analysis models was applied on these data, in order to take correlations of effect estimates coming from the same study into account. The influence of population (age, sex proportion [% male] and populations with various chronic diseases) and study characteristics (intervention type [only CWAT use, or a CWAT in combination with behaviour change strategies including goal setting, telephone support and/or individual or group counselling] and intervention duration) on the intervention effect was evaluated using a mixed effects meta-regression analysis. Here, the effects of these covariates were studied as fixed effects in the model. Tests for overall effect as well as pairwise comparisons among populations were performed. For the latter, adjustments for all other effects and corrections for multiple comparisons were used. Studies with missing values regarding the covariates were not included into the analysis. The beta estimate refers to the change in number of steps per day for a unit change in the characteristics. Sensitivity analyses were performed to assess heterogeneity of the studies and to evaluate the robustness of the results. Each study was individually removed to evaluate the effect of that study on the summary estimates. Publication bias was assessed using funnel plots and the Egger test (Appendix III). The effect of heterogeneity of each summary effect size was quantified using a chi-squared test and the I^2^ statistic, in which the boundary limits 25, 50, and 75% were designated as a low, moderate, and high heterogeneity value [31].

## Results

**Fig. 1:**
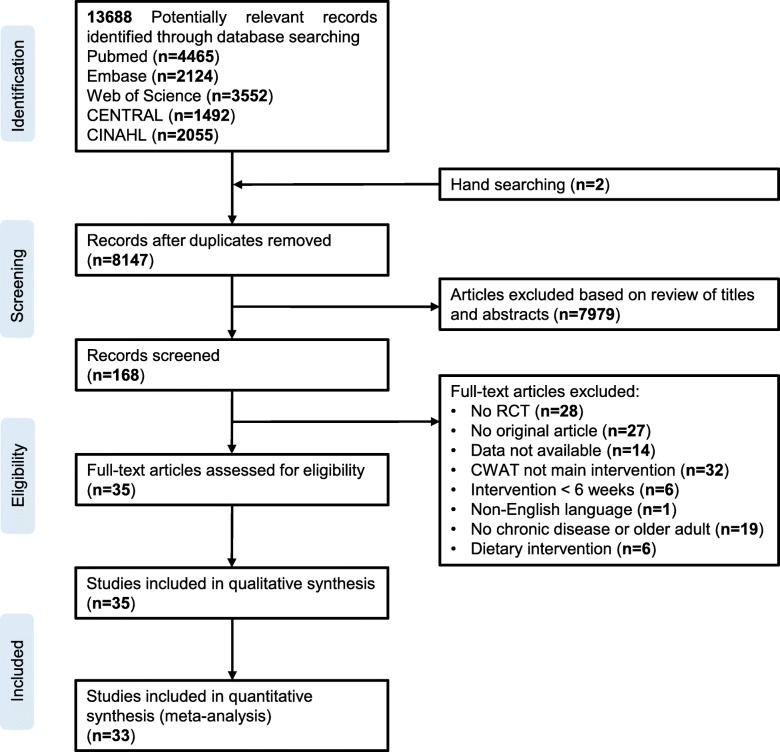
Flow diagram of the study selection process

The search strategy identified 13,688 potentially relevant studies of which 8147 after deduplication (Fig. 1). Thirty-five full-text articles fulfilled the inclusion criteria and were included in qualitative (*n* = 35) and quantitative (*n* = 33) synthesis (Table 1.). Studies included were published over a 14-year period from 2004 to 2018. All studies were written in English and performed in 13 different countries predominantly originating from the United States (*n* = 10). The included studies consisted of populations with various chronic diseases including chronic respiratory diseases (*n* = 7) [32–38], impaired glucose tolerance or T2DM (*n* = 12) [39–50], CVD (*n* = 6) [51–56], overweight/obesity (*n* = 3) [57–59], cognitive disorders (n = 1) [60] and sedentary older adults (n = 6) [61–65].

**Table 1:** Characteristics of included studies

Study	Population	Intervention duration (wk)	Dropout rate (%)	No. of participants	Intervention	CWAT type	Outcome parameter
**Altenburg 2015**	COPD (62 ± 1^a^ years; sex m/f: 102/53)	64	30	78	I: Goal setting, motivational interviewing techniques (five individual 30 min counselling sessions) and pedometer use for feedback and motivation	Yamax Digiwalker SW-200	Physical activity: - Steps/day
				77	C: Usual care		
**Araiza 2006**	T2DM (50 ± 10 years)	6	0	15	I: Pedometer use and instructed to walk 10.000 steps/day on 5 or more days of the week	Yamax Digiwalker SW-701	Physical activity: - Steps/day Cardiometabolic risk: - Body mass index - Waist circumference - Body fat percentage - Systolic blood pressure - Diastolic blood pressure - HbA1c - Fasting glucose - Fasting insulin - HOMA-IR - Triglycerides - Total cholesterol - HDL cholesterol - LDL cholesterol
				15	C: Pedometer use and instructed to maintain normal activity habits		
**Armit 2009**	Sedentary older adults (50–59 years: *n* = 78, 60–70 years: *n* = 58; sex m/f: 40/51)	12	0	46	I: Physical activity advice provided during a 30 min behaviour change counselling session (Transtheoritical model) and three follow-up telephone calls, pedometer use, goal setting and motivational interviewing techniques	–	Cardiometabolic risk: - Body mass index - Systolic blood pressure - Diastolic blood pressure - Resting heart rate
				45	C: Usual care (3-5 min of brief verbal physical activity advice and written information)		
**Baker 2008**	Overweight/Obesity (49.3 ± 8.8 years; sex m/f: 63/16)	12	20	39	I: Pedometer-based walking program with consultations based on the Transtheoretical Model of exercise behaviour change, goal setting (3000 steps/day above baseline)	Omron HJ-109E Step-O-Meter	Physical activity: - Steps/day Cardiometabolic risk: - Body weight - Body mass index - Waist circumference - Hip circumference - Waist-to-hip ratio - Body fat percentage - Systolic blood pressure - Diastolic blood pressure - Resting heart rate - Total cholesterol - HDL cholesterol - LDL cholesterol
				40	C: Maintain usual activity levels		
**Bjorgaas 2008**	T2DM (58.9 ± 10.5 years; sex m/f: 31/17)	26	31	23	I: Pedometer use, goal setting, physical activity calendar, encouraged to increase targeted number of steps	Yamax Digiwalker ML AW-320	Cardiometabolic risk: - Body weight - Systolic blood pressure - Diastolic blood pressure - HbA1c - Fasting glucose - Triglycerides - Total cholesterol - HDL cholesterol - Peak oxygen uptake
				25	C: Encouraged to increase daily time spent walking		
**Bond 2014**	Overweight/Obesity (46.0 ± 8.9 years; sex m/f: 10/65)	6	6	40	I: Individual face-to-face counseling sessions (6 sessions lasted 30–45 min) based on theoretical constructs from the Transtheoretical Model, Theory of Planned Behaviour, Social Cognitive Theory, and Self-Determination Theory; goal setting, pedometer use, problem solving, action planning, review of progress	–	Physical activity: - Steps/day
				35	C:Standard preoperative care. Surgeons and members of the surgical team advised participants to adopt an active lifestyle and engage in walking exercise and other similar activities		
**Butler 2009**	CVD (63.8 ± 10.8 years; sex m/f: 83/27)	26	26	55	I: Pedometer use, step calendar, four telephone calls, behavioural counselling, goal setting, generic physical activity information brochures	Yamax Digiwalker 700B	Cardiometabolic risk: - Body mass index - Waist circumference
				55	C: Generic physical activity information brochures		
**Coelho 2017**	Asthma (45.9 ± 16.7 years; sex m/f: 5/32)	12	19	20	I: Individual standardized educational session, Pedometer use, step based physical activity prescription, goal setting (steps taken during the previous week plus 1000 steps)	Yamax Digiwalker SW-200	Physical activity: - Steps/day
				17	C: Individual standardized educational session		
**Croteau 2007**	Sedentary older adults (72.9 ± 8.8 years; sex m/f: 32/115)	12	14	79	I: Behaviour change counselling session (Social cognitive theory), pedometer use, goal setting, select strategies for increasing daily step counts, review pedometer usage and discuss procedures for keeping a step calendar and monthly group sessions	Yamax Digiwalker SW-200	Physical activity: - Steps/day
				68	C: Instructed to continue usual activity habits		
**De Blok 2006**	COPD (64.1 ± 11.1 years; sex m/f: 9/12)	10	24	10	I: Behaviour change counselling programme and pedometer use for feedback and motivation	Yamax Digiwalker SW-200	Physical activity: - Steps/day
				11	C: Usual care (regular pulmonary rehabilitation programme)		
**De Greef 2010**	T2DM (35–54 years: n = 6, 55–75 years: n = 35; sex m/f: 28/13)	12	10	20	I: cognitive-behavioural group programme (5 sessions of 90 min), pedometer use	Yamax Digiwalker SW-200	Physical activity: - Steps/day Cardiometabolic risk: - Body weight - Body mass index - Systolic blood pressure - Diastolic blood 4pressure - HbA1c - Total cholesterol
				21	C: Usual care and one single group education on the effects of physical activity on diabetes care		
**De Greef 2011 (1)**	T2DM (67.4 ± 9.3 years; sex m/f: 47/20)	12	4	21	I1: Pedometer and diary use, goal setting, three 90 min group counselling sessions (based on the cognitive behavioural therapy, the Diabetes Prevention Program, the First Step Program and motivational interviewing) by a clinical psychologist	Yamax Digiwalker SW-200	Physical activity: - Steps/day Cardiometabolic risk: - Body mass index - Waist circumference - HbA1c - Fasting glucose - Total cholesterol
				22	I2: Pedometer and diary use, goal setting, three individual 15 min face-to-face consultations with a clinical psychologist		
				24	C: General care from the general practitioner		
**De Greef 2011 (2)**	T2DM (62 ± 9 years; sex m/f: 64/28)	26	4	60	I: Face-to-face session (based on the cognitive behavioural therapy, the Diabetes Prevention Program, the First Step Program and motivational interviewing), pedometer and diary use, 24-week telephone support programme	Yamax Digiwalker SW-200	Physical activity: - Steps/day
				32	C: General care from the general practitioner		
**Diedrich 2009**	T2DM (54.2 ± 11.6 years sex m/f: 18/35)	12	38	27	I: Diabetes Self-Management Education Program, *Manpo-Kei* book; pedometer use, record pedometer readings according to instructions in the book	Yamax Digiwalker SW-200	Cardiometabolic risk: - Body fat percentage - Systolic blood pressure - Diastolic blood pressure - HbA1c
				26	C: Diabetes Self-Management Education Program		
**Duscha 2018**	CVD (62.3 ± 8.3 years; sex m/f: 19/6)	12	22	16	I: CWAT use, exercise prescription by daily step count, health coaching (health related recommendations, planning, motivation and sending educational material via email and text messages)	Fitbit Charge	Physical activity: - Steps/day Cardiometabolic: - Peak oxygen uptake
				9	C: Usual care		
**Engel 2006**	T2DM (62.4 ± 7.7 years; sex m/f: 28/26)	26	7	24	I: Coaching (Diabetes-related education, goal setting, supportive/motivational strategies, behaviour change strategies and psychosocial support by telephone or face-to-face contact) and pedometer use	Yamax Digiwalker-700	Cardiometabolic: - Body weight - Body mass index - Waist circumference - Systolic blood pressure - Diastolic blood pressure - HbA1c
				30	C: Coaching (Diabetes-related education, goal setting, supportive/motivational strategies, behaviour change strategies and psychosocial support by telephone or face-to-face contact)		
**Hospes 2009**	COPD (62.2 ± 8.6 years; sex m/f: 21/14)	12	10	18	I: Customized exercise counselling programme (5 sessions), goal setting and pedometer use	Digiwalker SW-200	Physical activity: - Steps/day
				17	C: Usual care		
**Houle 2011**	CVD (58.5 ± 8.5 years; sex m/f: 51/14)	52	22	32	I: Pedometer use, diary, physical activity, goal setting (target of 3000 steps per day increment in physical activity counselling (social cognitive theory) by a clinical nurse specialist	Digiwalker SW-200	Physical activity: - Steps/day Cardiometabolic: - Waist circumference - Systolic blood pressure - Diastolic blood pressure - Resting heart rate - Fasting glucose - Triglycerides - LDL cholesterol - HDL cholesterol
				33	C: Usual care		
**Kaminsky 2013**	CVD (56.0 ± 9.2 years; sex m/f: 14/4)	8	36	10	I: Recommended to obtain a minimum of 30–40 min/day moderate-to-vigorous physical activity on days they did not attend cardiac rehabilitation, daily step count goals to increase by 10% of baseline step per day	NL-1000 New-lifestyles	Physical activity: - Steps/day
				8	C: Usual care		
**Kawagoshi 2015**	COPD (74.6 ± 8.4 years; sex m/f: 24/3)	52	31	12	I: Multidisciplinary home-based physical rehabilitation program, pedometer use, goal of 8000 steps per day, staff gave verbal reinforcement	Kenz Lifecorder EX	Cardiometabolic: - Body mass index
				15	C: Multidisciplinary home-based physical rehabilitation program		
**Kempf 2018**	Overweight/obese (45.4 ± 10.0 years; sex m/f: 81/99)	12	16	58	I1: Pedometer use, weekly care calls from trained coaches (information about overweight or obesity-related diseases like type 2 diabetes, healthy diet, physical activity, and coping strategies for lifestyle changes), participants were motivated to achieve individual goals using mental motivation program	SmartLAB walk P+	Cardiometabolic risk: - Body weight - Body mass index - Waist circumference - Systolic blood pressure - Diastolic blood pressure - HbA1c - Triglycerides - Total cholesterol - HDL cholesterol - LDL cholesterol
				61	I2: Pedometer use		
				61	C: Routine care		
**Kooiman 2018**	T2DM (56.4 ± 11.3 years; sex m/f: 38/34)	13	8	40	I: Usual care, activity tracker and access to online self-tracking (eHealth) program	Fitbit Zip	Cardiometabolic risk: - Body weight - Body mass index - Waist circumference - Hip circumference - Waist-to-hip ratio - HbA1c
				32	C: Usual care		
**Mendoza 2015**	COPD (68.7 ± 8.5 years; sex m/f: 62/40)	12	5	52	I: Pedometer use, diary with step count, goal setting	Tanita PD724 Triaxial pedometer	Physical activity: - Steps/day
				50	C: Counselling to increase physical activity (to walk at least 30 min/day)		
**Nolan 2017**	COPD (68 ± 9 years; sex m/f: 110/42)	26	26	76	I: Usual care (physical rehabilitation programme), pedometer use, goal setting and step count diary	Yamax Digiwalker CW700	Physical activity: - Steps/day
				76	C: Usual care (physical rehabilitation programme)		
**Rowley 2017**	Sedentary older adults (67.3 ± 6.3 years; sex m/f: 135/35)	12	24	51	I1:Pedometer use, given the goal to increase daily step count by 10% each week, when 10.000 steps per day were reached they were instructed to maintain 10.000 steps per day, incentives	Omron HJ-720ITC	Physical activity: - Steps/day
				62	I2: Pedometer use, interactive website with key strategies to increase PA systematically in older adults including education and goal setting, self-regulation, and frequent feedback and rewarding, incentives		
				57	C: Maintain usual activity levels		
**Suboc 2014** [1]	Sedentary older adults (63.1 ± 7.3 years; sex m/f: 71/36)	12	6	41	I1: Pedometer use, goal setting (10% above baseline each week to reach 10.000 steps/day)	Omron HJ-720ITC	Physical activity: - Steps/day Cardiometabolic risk: - Body weight - Body mass index - Waist circumference - Systolic blood pressure - Diastolic blood pressure - Resting heart rate - Fasting glucose - Fasting insulin - Triglycerides - Total cholesterol - HDL cholesterol - LDL cholesterol
				36	I2: Pedometer use combined with interactive website intervention (frequent feedback, motivational messages, self-regulation, goal setting, education and practice in realistic behavioural change strategies, rewarding)		
				30	C: Maintain usual activity levels		
**Suboc 2014** [2]	Sedentary older adults (63.0 ± 7.3 years; sex m/f: 35/67)	12	6	35	I1: Pedometer use, goal setting (10% above baseline each week to reach 10.000 steps/day)	Omron HJ-720ITC	Physical activity: - Steps/day Cardiometabolic risk: - Body weight - Body mass index - Waist circumference - Systolic blood pressure - Diastolic blood pressure - Resting heart rate
				28	I2: Pedometer use combined with interactive website intervention (frequent feedback, motivational messages, self-regulation, goal setting, education and practice in realistic behavioural change strategies, rewarding)		
				39	C: Maintain usual activity levels		
**Ter Hoeve 2018**	CVD (59.0 ± 8.5 years; sex m/f: 260/64)	64	34	161	I: Pedometer use, 3 face-to-face group PA counseling sessions consisted of information about health behavior, self-monitoring, goal setting, feedback, barrier identification and relapse prevention	Digiwalker SW-200	Physical activity: - Steps/day
				163	C: Usual care		
**Tudor-Locke 2004**	T2DM (52.7 ± 5.2 years; sex m/f: 26/21)	16	22	24	I: First Step behavioural modification program: Behaviour modification program based on the theoretical principles of self-efficacy and social support, the common clinical practices of goal setting, pedometer use and a calendar for self-monitoring and feedback	Digiwalker SW-200	Physical activity: - Steps/day Cardiometabolic: - Body weight - Body mass index - Waist circumference - Hip circumference - Systolic blood pressure - Diastolic blood pressure - Resting heart rate - HbA1c - Fasting glucose - Fasting insulin - Triglycerides - Total cholesterol - LDL cholesterol - HDL cholesterol
				23	C: Usual care		
**Van Dyck 2013**	T2DM (62 ± 9 years; sex m/f: 63/29)	52	0	60	I: Pedometer use, face-to-face session, telephone support and goal setting.	Digiwalker SW-200	Physical activity: - Steps/day Cardiometabolic: - Body weight - Body mass index - Waist circumference - Systolic blood pressure - HbA1c - Fasting glucose - Triglycerides - Total cholesterol - LDL cholesterol - HDL cholesterol
				32	C: Usual care		
**Vidoni 2016**	Cognitive impairment related to Alzheimer’s disease (72.3 ± 5.2 years; sex m/f: 12/9)	16	43	9	I: Pedometer use, goal setting (increase goal steps 20% each week and maintaining in weeks 7 and 8), bi-weekly phone calls to encourage physical activity	Fitbit Zip	Physical activity: - Steps/day
				12	C: Masked pedometers		
**Vogel 2017**	CVD (62.8 ± 9.5 years; sex m/f: 29/0)	12	0	13	I: Pedometer use, goal setting	Polar loop	Cardiometabolic: - Work rate
				16	C: Usual care		
**Yamada 2012**	Sedentary older adults (75.7 ± 6.8 years; sex m/f: 42/40)	26	6	40	I: Pedometer-based behavioural change program consisted of goal setting (increase daily steps by 10% each month), self-monitoring and feedback	Yamax Powerwalker EX-510	Physical activity: - Steps/day
				42	C: Maintain usual activity levels		
**Yates 2009**	Impaired glucose tolerance (65.5 ± 9.0 years; sex m/f: 37/21)	52	18	29	I: Group-based education program (PREPARE program), pedometer use, goal setting (increase activity levels by at least 3000 steps/day)	Digiwalker SW-200	Physical activity: - Steps/day Cardiometabolic: - Fasting glucose
				29	C: Usual care		
**Yates 2010**	Impaired glucose tolerance (65.0 ± 8.9 years; sex m/f: 33/17)	52	24	24	I: Single session group-based education program (PREPARE program), pedometer use, goal setting (increase activity levels by at least 3000 steps/day)	NL-800 New-lifestyles	Physical activity: - Steps/day Cardiometabolic: - Body weight - Body mass index - Fasting glucose - Triglycerides - Total cholesterol - HDL cholesterol
				26	C: Usual care

### Risk of bias

**Fig. 2:**
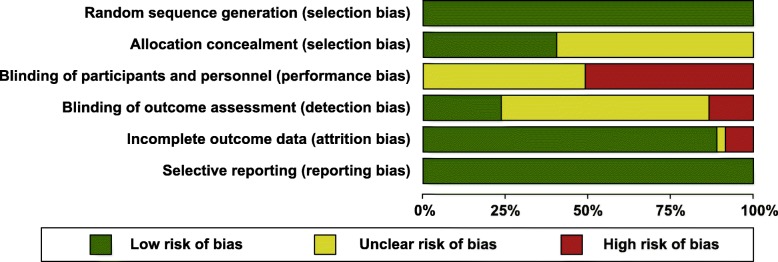
Risk of bias graph for included studies (n = 35)

Among the 35 included studies, several increased risks of bias were assessed. Fifteen studies met five (*n* = 5) [37, 42, 57, 59, 65] and four (*n* = 10) [18, 33, 38, 41, 49, 53, 55, 58, 64, 66] of the six risk of bias criteria. Eighteen studies met three criteria [32, 34–36, 39, 40, 43–45, 47, 50–52, 56, 60–63] and two studies met only two criteria [48, 54]. Although all studies reported appropriate random sequence allocation, insufficient information regarding the procedures used to conceal the allocation of the different trial arm(s) was provided in 21 of the 35 studies (Fig. 2). The largest risk of bias was with regard to the performance bias (lack of blinding of participants and personnel) and detection bias (minimal blinding of those assessing outcomes). All included studies were unable to blind the participants and the study personnel/physicians (*n* = 18) or to adequately report blinding (*n* = 17). Only eight studies blinded the outcome assessors, whereas the majority of the studies did not adequately report blinding of the outcome assessors (*n* = 22). Most of the studies provided complete outcome data (*n* = 31), whereas three studies had a high risk of attrition bias [38, 54, 60] due to incomplete outcome data and only one study reported insufficient information [48].

### Study characteristics

The CWAT-based interventions evaluated in the included studies varied considerably in intervention duration (mean ± SD; 22 ± 17 weeks; range: 6–64 weeks). In addition, various studies evaluated the effect of a multi-component intervention on physical activity and cardiometabolic health, such as CWAT use with individual counselling and goal setting (*n* = 10) and CWAT use with group counselling (*n* = 3) [41, 49, 50]. The majority of the interventions consisted of CWAT use and individual counselling (*n* = 22) of which five interventions also used additional telephone calls [48, 51, 59–61]. Control conditions varied between studies and consisted of usual care (*n* = 21) [32, 34–36, 38, 41–43, 46–50, 52–56, 58, 59, 61], maintaining normal physical activity levels (*n* = 7) [39, 57, 62–66], encouragement to increase daily physical activity (n = 2) [37, 40], generic information brochures (*n* = 1) [51], education sessions (n = 2) [33, 44], coaching sessions (n = 1) [45] and one control group wore masked CWAT devices (n = 1) [60]. Furthermore, the average dropout rate among the studies was 17% (range: 0–43%) of which four studies had a 100% participants completion rate.

### Population characteristics

The included studies evaluated a total of 2858 participants (intervention: *n* = 1567; control: *n* = 1291) of which 529 participants with chronic respiratory diseases (492 with chronic obstructive pulmonary disease; COPD), 704 participants with T2DM, 571 participants with CVD, 334 participants with overweight/obesity, 21 participants with Alzheimer’s disease and 699 sedentary older adults. Their mean age was 61.3 ± 7.3 years (range: 45.4–75.7 years) whereas two studies did not provide information of the average population age [41, 61]. Overall, 60% (range: 13–89%) of the participants were male and one study included only male participants. Twenty-five studies reported daily step counts and most of the participants in the intervention (5105 ± 2591 steps/day; range: 2031–9003 steps/day) and control (5149 ± 2751 steps/day; range: 2334–7539 steps/day) groups were relatively physically inactive at baseline.

### Physical activity

**Fig. 3:**
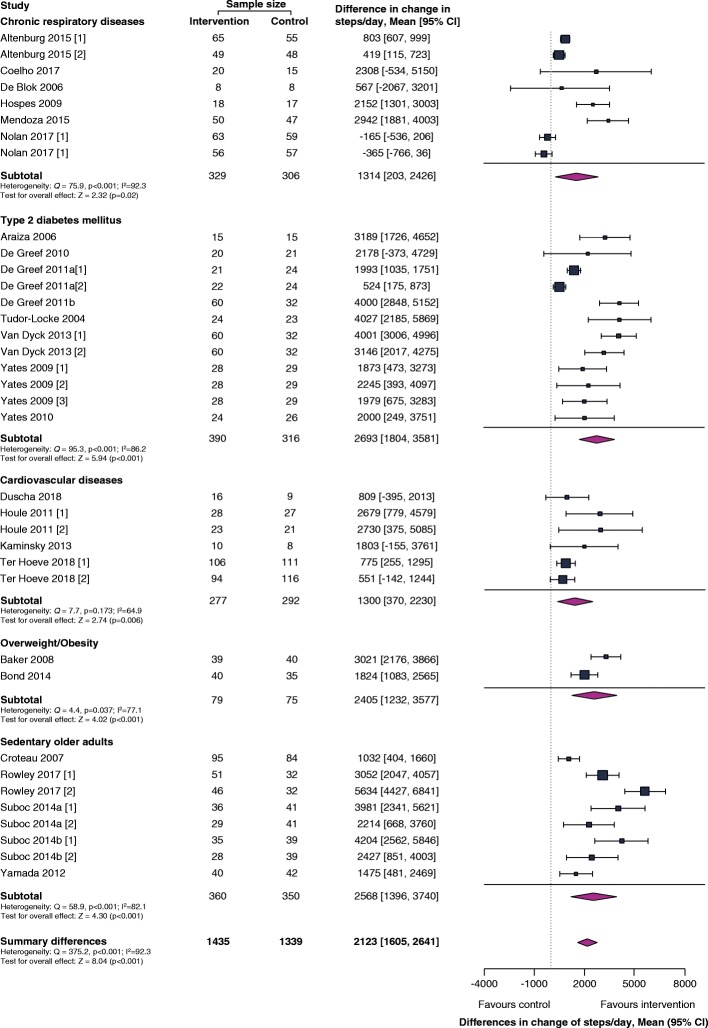
Forest plot of mean differences of physical activity (steps/day) from CWAT-based behaviour change interventions, compared to control groups. Abbreviations: CI: confidence interval, *I*^2^: the variation in pooled effect size attributable to heterogeneity within that group

Thirty-three of the studies included data on mean differences of physical activity, expressed as steps per day, and were included in the meta-analyses. Overall, participants with chronic diseases assigned to an intervention group significantly increased their physical activity (step count) by 2123 steps per day more than individuals from control groups (95% confidence interval [CI]: [1605, 2641] steps/day; *p* < 0.001). Subgroup analyses showed a significantly increased physical activity above baseline in all groups with a chronic disease, including chronic respiratory diseases (+ 1314 steps/day; 95% CI: [203, 2426] steps/day; *p* = 0.020), T2DM (+ 2693 steps/day; 95% CI: [1804, 3581] steps/day; *p* < 0.001), CVD (+ 1300 steps/day; 95% CI: [370, 2230] steps/day; *p* = 0.006), overweight/obesity (+ 2405 steps/day; 95% CI: [1232, 3577] steps/day; *p* < 0.001) and sedentary older adults (+ 2568 steps/day; 95% CI: [1396, 3740] steps/day; *p* < 0.001), compared to the control groups Fig. 3. However, these results were statistically heterogeneous with respect to the overall effect (*Q* = 375.2, *p* < 0.001; I^2^ = 92) as well as the results from individual subgroups including chronic respiratory diseases (*Q* = 75.9, p < 0.001; I^2^ = 97), T2DM (*Q* = 95.3, p < 0.001; I^2^ = 86), overweight/obesity (*Q* = 4.4, *p* = 0.037; I^2^ = 77) and sedentary older adults (*Q* = 58.9, p < 0.001; I^2^ < 82). In addition, significant publication bias was found in subgroup analyses for patients with CVD (*p* = 0.012), overweight/obesity (p = 0.037) and sedentary older adults (*p* = 0.004). Since only 1 study was found regarding cognitive diseases, this study was not included into the meta-analysis. No significant improvement in physical activity was found in patients with Alzheimer’s disease after an 8-week intervention period.

### Association between physical activity and participant and intervention characteristics

Meta-regression analysis showed that all participant characteristics were significantly associated with increased physical activity among individuals from the intervention groups. With respect to the participant characteristics, sex proportion (β = 41.0; *p* = 0.039) and CWAT users of a younger age (β = − 189.3; *p* = 0.007) were significantly associated with a higher increase in physical activity. In addition, patients with chronic respiratory diseases or CVD had a significantly lower increase in physical activity, compared to other populations with chronic diseases (*p* < 0.001). Furthermore, no significant association was found between the intervention duration (β = − 7.2; *p* = 0.951), intervention type (β = − 424.4; *p* = 0.528) and physical activity.

### Physical activity and cardiometabolic health

Participants from the intervention groups significantly decreased their waist circumference (− 0.99 cm; 95% CI: [− 1.48, − 0.50] cm; *p* < 0.001), systolic BP (− 3.79 mm Hg; 95% CI: [− 4.53, − 3.04] mm Hg; p < 0.001) and LDL cholesterol concentration (− 5.70 mg/dl; 95% CI: [− 9.24, − 2.15] mg/dl; *p* = 0.002) more than individuals from the control group (Table 2). In addition, both waist circumference (*Q* = 20.0, *p* = 0.40; I^2^ = 12), systolic BP (*Q* = 7.5, *p* = 0.98; I^2^ < 0.001) and LDL cholesterol concentration (*Q* = 9.0, *p* = 0.44; I^2^ < 0.001) were all homogeneous results and no significant publication bias was found. Furthermore, the study by Vogel et al. evaluated changes in physical fitness after a 6-week post-rehabilitation period and showed a significant increased peak workload in the CWAT intervention group (from 185 ± 55 to 192 ± 54 W [+ 27 W]; *p* < 0.001), compared to the control group (from 186 ± 52 to 169 ± 44 W [− 17 W]).

**Table 2:** Effects of CWAT-based behaviour change strategies on cardiometabolic health including anthropometrics, cardiovascular health and biochemical parameters

Characteristics	No. of studies	No. of participants (intervention/control)	Preintervention mean (SD)		Mean change (95% confidence interval)	p-value
			Intervention	Control		
**Anthropometrics**						
Body weight (kg)	11	582/570	90.6 (18.3)	88.4 (18.3)	−0.35 [−0.84, 0.13]	0.15
Body mass index (kg/m^2^)	16	730/733	30.2 (4.9)	30.5 (5.6)	−0.05 [−0.20, 0.11]	0.56
Waist circumference (cm)	13	715/715	103.6 (13.4)	103.3 (14.7)	−0.99 [−1.48, − 0.50]	**< 0.001**
**Cardiovascular**						
Systolic blood pressure (mm Hg)	13	655/654	133.1 (20.0)	131.7 (18.2)	−3.79 [−4.53, −3.04]	**< 0.001**
Diastolic blood pressure (mm Hg)	12	535/590	77.3 (13.9)	77.4 (12.0)	0.12 [−1.23, 1.46]	0.87
Resting heart rate (bpm)	6	295/320	68.5 (11.6)	67.4 (10.5)	−1.92 [−3.96, 0.13]	0.07
**Biochemical**						
HbA1c (%)	11	462/424	7.0 (1.4)	7.1 (1.4)	−0.07 [−0.16, 0.01]	0.08
Fasting glucose (mg/dl)^a^	9	471/431	124.1 (40.1)	116.9 (37.3)	−1.67 [−5.82, 2.49]	0.43
Triglycerides (mg/dl)^a^	8	452/414	151.7 (96.6)	149.0 (103.1)	−1.11 [−8.03, 5.81]	0.75
Total cholesterol (mg/dl)^a^	11	483/459	183.9 (44.2)	187.6 (49.4)	−1.48 [−7.48, 4.51]	0.63
HDL cholesterol (mg/dl)^a^	9	484/448	46.7 (16.1)	48.0 (18.0)	0.29 [−1.13, 1.72]	0.69
LDL cholesterol (mg/dl)^a^	6	408/364	98.4 (39.8)	102.8 (45.3)	−5.70 [−9.24, −2.15]	**0.002**

Abbreviations: LDL: low-density lipoprotein, HDL: High-density lipoprotein

^a^Blood parameters were converted to the same unit (from mmol/l to mg/dl), including triglycerides (divide by 0.0112), total cholesterol, HDL cholesterol, LDL cholesterol (divide by 0.02586) and glucose (divide by 0.05551) concentration

## Discussion

This review systematically evaluated the efficacy of CWAT-based interventions to promote physical activity levels and improve cardiometabolic health in sedentary older adults and patients with chronic diseases, including chronic respiratory diseases, T2DM, CVD, overweight/obesity and cognitive disorders. To the best of our knowledge, this is the first systematic review and meta-analysis that evaluates the impact of CWATs on physical activity and cardiometabolic health in populations with various chronic diseases. In addition, these results are solely based on randomised controlled trials and objectively measured physical activity (number of steps per day). In general, individuals with a CWAT increased their physical activity level and this may be associated with improvements in cardiometabolic health including waist circumference, systolic BP and LDL cholesterol concentration.

Physical activity levels increased by 2123 steps per day. This is comparable to 20 min or 1.5 km of walking. These results are consistent with findings from other meta-analyses showing an increased physical activity among outpatient adults (+ 2500 steps/day) [14] and patients with T2DM (+ 1822–2042 steps/day) [21, 22]. In addition, de Vries et al. showed that behavioural phyical actvity interventions with an activity monitor increase physical activity (both steps/day and moderate-to-vigorous physical activity) in adults with overweight and obesity [20]. Armstrong et al. found that pedometer-based physical activity promotion increased steps per day (+ 1000 steps/day) when it is used as an intervention alone or alongside pulmonary rehabilitation [67]. These studies all confirm that a CWAT-only, or included in a multi-component intervention, have a positive effect in favor of the interventions groups. Here, Dwyer et al. and Jefferis et al. both found an association between all-cause mortality and step count where every increment of 1000 steps per day led to a 6 and 14% risk reduction, respectively [68, 69]. In addition, the NAVIGATOR study, which consisted of 9000 individuals with high cardiovascular risk or impaired glucose tolerance, has shown that for every additional 2000 steps/day the risk of developing cardiovascular events decreased by 10%, T2DM by 5.5%, and the metabolic syndrome risk score redcued by 0.29 *z*-score [70–72]. In general, CWAT-users included in this meta-analysis increased their step count from 5100 steps per day at baseline to 7200 after the intervention period. Consequently and in accordance with Lee et al., who obeserved that hazard ratios of all-cause mortality declined progressively with higher mean steps per day until approximately 7500 steps/day after which they leveled in older adult women [73], this could indicate a substantial reduction in all-cause mortality risk and the development of chronic diseases. As such, our findings are relevant for the general population and for those who cannot participate in high-intensity physical activities.

Meta-regression analysis showed that both patients with COPD and CVD exhibited the lowest increase in physical activity among all populations with chronic diseases included in this systematic review. This might be due to airflow and cardiac limitations and most of these studies were performed after a rehabilitation period where baseline physical activity levels were reduced. These findings were also found by Amstrong et al.*,* who showed that COPD patients with greater baseline physical activity levels (> 4000 steps/day) had greater improvements in steps per day. These results can be confirmed by our results since the baseline physical activity levels of COPD patients in our meta-analysis were 3335 steps per day. In addition, meta-regression analysis showed no additional effect of behaviour change strategies (goal setting, group counselling, individual counselling or telephone support) on physical activity levels. This is in contrast with a systematic review that showed a positive effect for CWATs, even when interventions were separated into CWAT-only and multifaceted interventions [74]. However, a larger effect size for interventions that were multifaceted was found compared to CWAT-only interventions. This suggests that CWATs can be effective on their own, but when combined with other behaviour change strategies, such as telephone support or group-based counselling, the improvement in physical activity is greater. However, it must be outlined that Brickwood et al. only included healthy adults and there was only a small number of studies in our meta-regression that included both a CWAT-only and CWATs as part of a multi-component intervention group. Therefore, more studies are warranted which include both a CWAT-only and a multi-component intervention group. Furthermore, patient characteristics were all independently associated with an increased number of step, which possibly explains the heterogeneity of the overall effect and subgroup analyses. For example, the obese population was younger of age compared to other populations and, as a result, propably showed a higher increase in physical activity. Therefore, caution is warranted when interpreting these results.

The increment of physical activity was associated with improvements in several cardiometabolic health parameters. The systolic BP was significantly decreased in the intervention groups. This finding is consistent with previous systematic reviews with respect to physical activity and blood pressure among outpatient [14] and healthy sedentary adults [75, 76]. It has been shown that a change of − 10 mm Hg in systolic BP reduces the risk of developing CVD by 17% and overall mortality by 13% [77]. In addition, a decrease in systolic BP of 2 mm Hg is associated with a reduction of 10% in stroke mortality and a 7% reduction in mortality originating from CVD [78]. Furthermore, a significantly reduced LDL cholsterol concentration was found when physical activity levels were increased among individuals with CWATs. A meta-analysis from The Cholesterol Treatment Trialists’ Collaboration showed that with the aid of statins the 5-year incidence of major vascular events reduced by about 20% and all-cause mortality by 12% per 40 mg/dl in LDL cholesterol concentration. However, since we showed a reduction of 6 mg/dl, medication has probably more beneficial effects rather than increasing physical activity [79]. In addition, most data regarding physical activity and serum lipids seem to indicate that regular physical activity or exercise training does not reduce LDL cholesterol concentration in a clinically relevant way [80, 81]. However, it has been suggested that regular physical activity may change the LDL cholesterol particle size, even when total LDL concentrations remain constant [82, 83]. We also found a significantly decreased waist circumference with the same magnitude (− 1.19 cm; 95% CI: [− 1.79, − 0.59] cm) as found in another published meta-analysis in which the effect of physical activity and dietery interventions on waist circumference was evaluated [11]. De Koning et al. have shown that a decrease of 1 cm in waist circumference reduces the relative risk of a CVD event by 2% [84]. In this way, CWAT devices can improve health related outcomes which are closely related to CVD.

Several limitations of the included studies were observed. First, studies with relatively small sample sizes and different intervention durations were included in the meta-analyses. In addition, this review reflected heterogeneous study designs which consisted of multiple components including CWAT use, goal setting, telephone calls, diary use, individual or group counselling, different therapists, face-to-face or written motivation and the frequency of the sessions. Therefore, the independent contribution of any of these components, or a combination of these factors, is difficult to establish. This probably explains the high heterogeneity of the physical activity results (Q = 375, *p* < 0.001; I^2^ = 92). However, since clinical and methodological diversity is always present in meta-analysis statistical heterogeneity is inevitable. Moreover, Higgens et al. reported that almost one third of meta-analyses have moderate to considerable heterogeneity [85]. Secondly, only eleven studies evaluated physical activty (steps/day) in combination with one or more of the cardiometabolic risk factors. In this way, no direct associations between increased physical acitivities (steps/day) and cardiometabolic risk factors could be made and no minimal amount of steps per day to improve cardiometabolic health could be determined. Thirdly, various CWAT devices were both used to objectively measure physical activity and as a motivational instrument. As a result, this may affect the control group by increasing their physical activity by the thought that they were being monitored. Finally, all studies only measured physical activity but did not take into account sedentary time. It has been shown that sedentary behaviour is a risk factor for cardiometabilic health and all-cause mortality, indepently of the amount of physical activity [86, 87]. Therefore, we recommend to include sedentary time, next to physical activity, in future studies.

## Conclusion

These results suggest that populations with chronic diseases significantly increase physical activity using CWATs only, or as part of a multi-component intervention, and improve their cardiometabolic health such as a reduced waist circumference, systolic BP and LDL cholesterol concentration.

## Supplementary information

**Additional file 1: Appendix I** Pubmed search.

**Additional file 2: Appendix II** Data extraction form.

**Additional file 3: Appendix III** Funnel plots.

## Data Availability

All data generated or analysed during this systematic review are included in this published article [and its supplementary information files].

## Abbreviations

CWAT: Consumer wearable activity tracker
T2DM: Type 2 diabetes mellitus
CVD: Cardiovascular diseases
WHO: World Health Organization
PRISMA: Preferred Reporting Items for Systematic reviews and Meta-Analysis
CENTRAL: Cochrane Central Register of Controlled Trials
BMI: Body Mass Index
BP: Blood pressure
HDL: High-density lipoprotein
LDL: Low-density lipoprotein
HbA1c: Blood glycated haemoglobin concentration
COPD: Chronic obstructive pulmonary disease
