# Supplementary material for: Can consumer wearable activity tracker-based interventions improve physical activity and cardiometabolic health in patients with chronic diseases? A systematic review and meta-analysis of randomised controlled trials
Source: Int J Behav Nutr Phys Act. 2020 May 11;17:57. doi: 10.1186/s12966-020-00955-2 (PMC7216601; doi:10.1186/s12966-020-00955-2)
Supplement: Supplementary file 1 — Additional file 1: Appendix I Pubmed search. [file 12966_2020_955_MOESM1_ESM.docx]

**Pubmed search**

*NCD Population*

"Cardiac Rehabilitation"[MeSH] OR "Cardiovascular Diseases"[MeSH] OR "Chronic Disease"[MeSH] OR Aortic Stenoses[tiab] OR Aortic valve disease*[tiab] OR Aortic Valve Stenos*[tiab] OR Arteriosclerosis[tiab] OR Atherogenesis[tiab] OR Atheroscleroses[tiab] OR Atherosclerosis[tiab] OR Cardiac Disease*[tiab] OR Cardiac dysfunction*[tiab] OR Cardiac rehabilitation*[tiab] OR Cardiomyopath*[tiab] OR Cardiovascular disease*[tiab] OR Cardiovascular rehabilitation*[tiab] OR Cardiovascular risk*[tiab] OR Cardiovascular Stroke[tiab] OR CHF[tiab] OR Chronic disease*[tiab] OR Chronic Illness*[tiab] OR Coronary Disease*[tiab] OR CVD[tiab] OR Heart Attack*[tiab] OR Heart Disease*[tiab] OR Heart dysfunction*[tiab] OR Heart Failure*[tiab] OR Heart Valve Disease*[tiab] OR Myocardial Disease*[tiab] OR Myocardial Failure*[tiab] OR Myocardial Infarction*[tiab] OR Myocardial Ischemia*[tiab] OR Myocardiopath*[tiab] OR Peripheral Angiopath*[tiab] OR Vascular Disease*[tiab] OR Ventricular outflow obstruction*[tiab] OR Arrhythm*[tiab] OR Atrial flutter*[tiab] OR Tachycard*[tiab] OR Tachyarrhythm*[tiab] OR Arterial disease*[tiab] OR "Lung diseases"[MeSH] OR Chronic Airflow Obstruction*[tiab] OR Chronic bronchitis[tiab] OR Chronic Obstructive Airway Disease*[tiab] OR Chronic obstructive pulmonary disease*[tiab] OR Chronic respiratory disease*[tiab] OR COAD[tiab] OR COPD[tiab] OR Emphysema*[tiab] OR Lung disease*[tiab] OR "Glucose Metabolism Disorders"[MeSH] OR Diabetes[tiab] OR Glucose Intolerance*[tiab] OR Glucose metabolism disorder*[tiab] OR Glucose tolerance*[tiab] OR Glucose Metabolic Disorder*[tiab] OR IDDM[tiab] OR Impaired fasting glucose[tiab] OR Prediabetes[tiab] OR Pre-diabetes[tiab] OR Prediabetic State*[tiab] OR "Dyslipidemias"[MeSH] OR Dyslipidemia*[tiab] OR Dyslipoproteinemia*[tiab] OR Dysmetabolic Syndrome X[tiab] OR Elevated Cholesterol*[tiab] OR High Cholesterol Level*[tiab] OR Hypercholesteremia*[tiab] OR Hypercholesterolemia*[tiab] OR Hyperlipemia*[tiab] OR Hyperlipidaemia[tiab] OR Hyperlipidemia*[tiab] OR Hypertriglyceridemia*[tiab] OR Lipemia*[tiab] OR Lipidemia*[tiab] OR Metabolic Cardiovascular Syndrome*[tiab] OR High Cholesterol[tiab] OR High Blood Pressure[tiab] OR Hypertension[tiab] OR Hyperglycemia*[tiab] OR Hyperinsulinemia[tiab] OR Insulin resistance[tiab] OR Insulin sensitivity[tiab] OR Metabolic syndrome*[tiab] OR "Overweight"[MeSH] OR Obes*[tiab] OR Overweight*[tiab] OR "Cerebrovascular Disorders"[MeSH] OR Apoplex*[tiab] OR Brain Infarction*[tiab] OR Brain Vascular Accident*[tiab] OR Cerebrovascular Accident*[tiab] OR CVA[tiab] OR Stroke*[tiab] OR Cerebrovascular disease*[tiab] OR Cerebral Ischemia*[tiab] OR Brain ischemia*[tiab] OR "Multiple sclerosis"[MeSH] OR Disseminated Scleros*[tiab] OR MS[tiab] OR Multiple Scleros*[tiab] OR "Neurocognitive disorders"[MeSH] OR Alzheimer Disease*[tiab] OR Alzheimer's Disease*[tiab] OR Cognition Disorder*[tiab] OR Cognitive Decline*[tiab] OR Cognitive Dysfunction*[tiab] OR Cognitive Impairment*[tiab] OR Cognitive defect*[tiab] OR Dement*[tiab] OR Neurocognitive Disorder*[tiab] OR Neurological Disorder*[tiab]

*Sedentary older adult population*

"Postmenopause"[MeSH] OR "Adult"[MeSH] OR Septuagenarian*[tiab] OR Nonagenarian*[tiab] OR Octogenarian*[tiab] OR Octagenarian*[tiab] OR Centenarian*[tiab] OR Centarian*[tiab] OR Supercentenarian*[tiab] OR Elder*[tiab] OR frail*[tiab] OR geriatri*[tiab] OR old age*[tiab] OR oldest old*[tiab] OR senior*[tiab] OR senium[tiab] OR very old*[tiab] OR older people[tiab] OR older subject*[tiab] OR older patient*[tiab] OR older age*[tiab] OR older adult*[tiab] OR older man[tiab] OR older men[tiab] OR older male*[tiab] OR older woman[tiab] OR older women[tiab] OR older female*[tiab] OR older population*[tiab] OR older person*[tiab] OR adult*[tiab] OR postmenopaus*[tiab]

*Consumer wearable activity tracker*

(((("Telemedicine"[MeSH] OR "Telerehabilitation"[MeSH] OR "Wearable electronic devices"[MeSH] OR Telemedicine[tiab] OR Telerehabilitation[tiab] OR remote rehabilitation[tiab] OR Mobile Health[tiab] OR mHealth[tiab] OR m-health[tiab] OR Pedometer*[tiab] OR Acceleromet*[tiab]))) AND ((Rehabilitation[tiab] OR Treatment*[tiab] OR Intervention*[tiab] OR Prevention[tiab]))) OR (((Fitness Tracker*[tiab] OR Activity Tracker*[tiab] OR Smart wearable*[tiab] OR Activity monitor*[tiab] OR Remote patient monitoring[tiab] OR Fitness device*[tiab] OR SenseWear[tiab] OR BodyMedia Fit[tiab] OR DirectLife[tiab] OR Fitbit[tiab] OR Garmin[tiab] OR Vivosmart[tiab] OR Jawbone[tiab] OR MisFit Shine[tiab] OR Nike FuelBand[tiab] OR Polar[tiab] OR Withings[tiab] OR Yamax[tiab] OR Bodybugg[tiab] OR Tomtom[tiab] OR Fitbug[tiab] OR Wahoo[tiab] OR Omron[tiab] OR Apple Watch[tiab] OR Actiwatch[tiab] OR Smart watch*[tiab] OR Smart wristband*[tiab])))

*Behaviour change*

"Health behavior"[MeSH] OR "Health promotion"[MeSH] OR "Leisure activities"[MeSH] OR "Motor activity"[MeSH] OR Physical Activit*[tiab] OR Health Behavior*[tiab] OR Healthy Behavior*[tiab] OR Health Behaviour*[tiab] OR Healthy Behaviour*[tiab] OR Health Promotion*[tiab] OR Leisure Activit*[tiab] OR Physical Exercis*[tiab] OR Measure activit*[tiab] OR Behavior modification*[tiab] OR Behaviour modification*[tiab] OR Behavioral modification*[tiab] OR behavioural modification*[tiab] OR Behavior change*[tiab] OR Behaviour change*[tiab] OR Motor Activit*[tiab] OR Sedentary[tiab] OR Leisure time[tiab] OR lifestyle modification*[tiab] OR lifestyle change*[tiab]

*Cardiometabolic health outcomes*

"Physical fitness"[MeSH] OR "Exercise tolerance"[MeSH] OR "Blood pressure"[MeSH] OR "Heart rate"[MeSH] OR "Body weights and measures"[MeSH] OR "Body constitution"[MeSH] OR "Cholesterol"[MeSH] OR "Fat body"[MeSH] OR "Anthropometry"[MeSH] OR Oxygen uptake[tiab] OR Oxygen consumption[tiab] OR VO2[tiab] OR Physical Fitness[tiab] OR Exercise tolerance[tiab] OR Weight[tiab] OR Blood Pressure[tiab] OR Diastolic Pressure[tiab] OR Systolic Pressure[tiab] OR Pulse Pressure[tiab] OR Steps[tiab] OR Step count[tiab] OR MVPA[tiab] OR Moderate to vigorous activit*[tiab] OR Moderate to vigorous intensit*[tiab] OR Energy Expenditure[tiab] OR Heart Rate*[tiab] OR Pulse Rate*[tiab] OR Walking distance[tiab] OR Body Composition*[tiab] OR Body Constitution*[tiab] OR Lipid profile*[tiab] OR Cholesterol[tiab] OR LDL[tiab] OR HDL[tiab] OR Insulin[tiab] OR Glucose[tiab] OR Body fat[tiab] OR Waist Circumference*[tiab] OR Body Measure*[tiab] OR Waist-Hip Ratio*[tiab] OR Waist-to-hip ratio*[tiab] OR Anthropometr*[tiab] OR Metabolic health[tiab] OR Health outcome*[tiab] OR BMI[tiab] OR body mass index[tiab]

**Embase search**

*NCD Population*

"Chronic Disease".ti,ab,kw. OR "Chronic Illness*".ti,ab,kw. OR "Cardiovascular disease*".ti,ab,kw. OR CVD.ti,ab,kw. OR "Vascular disease*".ti,ab,kw. OR Atheroscleros*.ti,ab,kw. OR Arterioscleros*.ti,ab,kw. OR Atherogenesis.ti,ab,kw. OR "Peripheral Angiopath*".ti,ab,kw. OR "Heart failure*".ti,ab,kw. OR "Myocardial Failure*".ti,ab,kw. OR CHF.ti,ab,kw. OR "Heart Disease*".ti,ab,kw. OR "Cardiac Disease*".ti,ab,kw. OR Cardiomyopath*.ti,ab,kw. OR "Myocardial Disease*".ti,ab,kw. OR Myocardiopathy.ti,ab,kw. OR Myocardiopathies.ti,ab,kw. OR "Myocardial Ischemia*".ti,ab,kw. OR "Myocardial infarction*".ti,ab,kw. OR "Cardiovascular Stroke".ti,ab,kw. OR "Heart Attack*".ti,ab,kw. OR "Heart infarction".ti,ab,kw. OR "Heart Valve Disease*".ti,ab,kw. OR "Valvular Heart Disease*".ti,ab,kw. OR "Ventricular outflow obstruction*".ti,ab,kw. OR "Aortic Valve Stenos*".ti,ab,kw. OR "Aortic Stenos*".ti,ab,kw. OR "Coronary Disease*".ti,ab,kw. OR "Coronary Heart Disease*".ti,ab,kw. OR "Cardiac Rehabilitation*".ti,ab,kw. OR "Cardiovascular rehabilitation*".ti,ab,kw. OR "Atrial flutter".ti,ab,kw. OR Tachycard*.ti,ab,kw. OR Tachyarrhythm*.ti,ab,kw. OR "Aortic valve disease*".ti,ab,kw. OR Arrhythm*.ti,ab,kw. OR exp chronic disease/ OR exp cardiovascular disease/ OR exp cardiovascular risk/ OR exp vascular disease/ OR exp atherosclerosis/ OR exp atherogenesis/ OR exp arteriosclerosis/ OR exp heart failure/ OR exp cardiomyopathy/ OR exp myocardial disease/ OR exp heart muscle ischemia/ OR exp heart infarction/ OR exp heart disease/ OR exp heart outflow tract obstruction/ OR exp aortic stenosis/ OR exp coronary artery disease/ OR exp heart rehabilitation/ OR exp aortic valve disease/ OR exp heart arrhythmia/ OR Lung disease*.ti,ab,kw. OR Chronic obstructive pulmonary disease*.ti,ab,kw. OR COPD.ti,ab,kw. OR COAD.ti,ab,kw. OR Chronic Airflow Obstruction*.ti,ab,kw. OR Chronic respiratory disease*.ti,ab,kw. OR Emphysema*.ti,ab,kw. OR Chronic bronchitis.ti,ab,kw. OR exp lung disease/ OR exp chronic respiratory tract disease/ OR exp emphysema/ OR exp chronic bronchitis/ OR Prediabetic State*.ti,ab,kw. OR Pre-diabetes.ti,ab,kw. OR Prediabetes.ti,ab,kw. OR Glucose metabolism disorder*.ti,ab,kw. OR Glucose Metabolic Disorder*.ti,ab,kw. OR Diabetes.ti,ab,kw. OR IDDM.ti,ab,kw. OR Glucose Intolerance*.ti,ab,kw. OR Glucose tolerance*.ti,ab,kw. OR Impaired fasting glucose.ti,ab,kw. OR exp "disorders of carbohydrate metabolism"/ OR exp glucose tolerance/ OR exp insulin sensitivity/ OR exp hyperinsulinism/ OR Hyperinsulinism.ti,ab,kw. OR Hyperinsulinemia.ti,ab,kw. OR Insulin Resistance.ti,ab,kw. OR Insulin sensitivity.ti,ab,kw. OR Metabolic syndrome*.ti,ab,kw. OR Dysmetabolic Syndrome X.ti,ab,kw. OR Metabolic Cardiovascular Syndrome*.ti,ab,kw. OR Dyslipidemia*.ti,ab,kw. OR Dyslipoproteinemia*.ti,ab,kw. OR Hyperlipemia*.ti,ab,kw. OR Hyperlipidaemia.ti,ab,kw. OR Hyperlipidemia*.ti,ab,kw. OR Lipidemia*.ti,ab,kw. OR Lipemia*.ti,ab,kw. OR Hypercholesterolemia*.ti,ab,kw. OR High Cholesterol Level*.ti,ab,kw. OR Elevated Cholesterol*.ti,ab,kw. OR Hypercholesteremia*.ti,ab,kw. OR Hypertriglyceridemia.ti,ab,kw. OR Hypertension.ti,ab,kw. OR High Blood Pressure.ti,ab,kw. OR exp dyslipidemia/ OR exp hyperlipoproteinemia/ OR exp hyperlipidemia/ OR exp hypercholesterolemia/ OR exp hypertriglyceridemia/ OR exp hypertension/ OR Obes*.ti,ab,kw. OR Overweight.ti,ab,kw. OR exp obesity/ OR exp intraabdominal fat/ OR exp overweight/ OR Apoplex*.ti,ab,kw. OR Brain Infarction*.ti,ab,kw. OR Brain Vascular Accident*.ti,ab,kw. OR Cerebrovascular Accident*.ti,ab,kw. OR CVA.ti,ab,kw. OR Stroke*.ti,ab,kw. OR Cerebral ischemia*.ti,ab,kw. OR Brain ischemia*.ti,ab,kw. OR Cerebrovascular disease*.ti,ab,kw. OR exp cerebrovascular accident/ OR exp cerebrovascular disease/ OR exp brain ischemia/ OR exp brain infarction/ OR Multiple scleros*.ti,ab,kw. OR Disseminated Scleros*.ti,ab,kw. OR MS.ti,ab,kw. OR exp multiple sclerosis/ OR Neurocognitive Disorder*.ti,ab,kw. OR Cognitive defect*.ti,ab,kw. OR Cognition Disorder*.ti,ab,kw. OR Dementia*.ti,ab,kw. OR Alzheimer Disease*.ti,ab,kw. OR Alzheimer's Disease*.ti,ab,kw. OR Cognitive dysfunction*.ti,ab,kw. OR Cognitive Impairment*.ti,ab,kw. OR Cognitive Decline*.ti,ab,kw. OR Neurological Disorder*.ti,ab,kw. OR exp cognitive defect/

*Sedentary older adult population*

Aged.ti,ab,kw. OR Postmenopaus*.ti,ab,kw. OR Septuagenarian*.ti,ab,kw. OR Nonagenarian*.ti,ab,kw. OR Octogenarian*.ti,ab,kw. OR Octagenarian*.ti,ab,kw. OR Centenarian*.ti,ab,kw. OR Centarian*.ti,ab,kw. OR Supercentenarian*.ti,ab,kw. OR Elder*.ti,ab,kw. OR old age*.ti,ab,kw. OR geriatri*.ti,ab,kw. OR oldest old*.ti,ab,kw. OR senior*.ti,ab,kw. OR very old*.ti,ab,kw. OR older people.ti,ab,kw. OR older subject*.ti,ab,kw. OR older patient*.ti,ab,kw. OR older age*.ti,ab,kw. OR older adult*.ti,ab,kw. OR older man.ti,ab,kw. OR older men.ti,ab,kw. OR older male*.ti,ab,kw. OR older woman.ti,ab,kw. OR older women.ti,ab,kw. OR older female*.ti,ab,kw. OR older population*.ti,ab,kw. OR older person*.ti,ab,kw. OR adult*.ti,ab,kw. OR exp postmenopause/ OR exp adult/ OR exp aging/

*Consumer wearable activity tracker*

Rehabilitation.ti,ab,kw. OR Treatment*.ti,ab,kw. OR Intervention*.ti,ab,kw. OR Prevention.ti,ab,kw.

**AND**

Acceleromet*.ti,ab,kw. OR Telemedicine.ti,ab,kw. OR Mobile Health.ti,ab,kw. OR mHealth.ti,ab,kw. OR m-health.ti,ab,kw. OR Telerehabilitation.ti,ab,kw. OR Tele-rehabilitation.ti,ab,kw. OR Remote Rehabilitation.ti,ab,kw. OR Pedometer.ti,ab,kw. OR Wearable Electronic Device*.ti,ab,kw. OR Wearable Device*.ti,ab,kw. OR Monitoring device*.ti,ab,kw.

**OR**

Fitness Tracker*.ti,ab,kw. OR Activity Tracker*.ti,ab,kw. OR BodyMedia Fit.ti,ab,kw. OR Sensewear.ti,ab,kw. OR DirectLife.ti,ab,kw. OR Fitbit.ti,ab,kw. OR Garmin.ti,ab,kw. OR Vivosmart.ti,ab,kw. OR Jawbone.ti,ab,kw. OR MisFit Shine.ti,ab,kw. OR Nike FuelBand.ti,ab,kw. OR Polar.ti,ab,kw. OR Withings.ti,ab,kw. OR Yamax.ti,ab,kw. OR Bodybugg.ti,ab,kw. OR Tomtom.ti,ab,kw. OR Fitbug.ti,ab,kw. OR Wahoo.ti,ab,kw. OR Apple Watch.ti,ab,kw. OR Smart wearables.ti,ab,kw. OR Wearable electronic device*.ti,ab,kw. OR Wearable technolog*.ti,ab,kw. OR Wearable sensor*.ti,ab,kw. OR Self-monitoring.ti,ab,kw. OR Self-tracking.ti,ab,kw. OR Activity monitoring.ti,ab,kw. OR Activity monitor*.ti,ab,kw. OR Remote patient monitoring.ti,ab,kw. OR Fitness device*.ti,ab,kw. OR Omron.ti,ab,kw. OR Actiwatch.ti,ab,kw. OR Smart watch*.ti,ab,kw. OR Smart wristband.ti,ab,kw. OR exp telerehabilitation/ OR exp activity tracker/ OR exp ambulatory monitoring/ OR exp self monitoring/

*Behaviour change*

Physical Activit*.ti,ab,kw. OR Health monitoring.ti,ab,kw. OR Health Behavior*.ti,ab,kw. OR Health Behaviour*.ti,ab,kw. OR Health Promotion*.ti,ab,kw. OR Leisure Activit*.ti,ab,kw. OR Physical Exercise*.ti,ab,kw. OR Measure activity.ti,ab,kw. OR Behavior Modification*.ti,ab,kw. OR Motor Activit*.ti,ab,kw. OR Sitting time.ti,ab,kw. OR Sedentary.ti,ab,kw. OR Leisure time.ti,ab,kw. OR Treatment*.ti,ab,kw. OR Lifestyle modification*.ti,ab,kw. OR Lifestyle change*.ti,ab,kw. OR exp physical activity/ OR exp health behavior/ OR exp behavior modification/ OR exp behavior therapy/ OR exp motor activity/ OR exp sitting/ OR exp sedentary lifestyle/

*Cardiometabolic health outcomes*

Oxygen uptake.ti,ab,kw. OR Oxygen consumption.ti,ab,kw. OR VO2.ti,ab,kw. OR Physical fitness.ti,ab,kw. OR Exercise tolerance.ti,ab,kw. OR Weight.ti,ab,kw. OR Blood pressure.ti,ab,kw. OR Diastolic Pressure.ti,ab,kw. OR Systolic Pressure.ti,ab,kw. OR Pulse Pressure.ti,ab,kw. OR Step*.ti,ab,kw. OR MVPA.ti,ab,kw. OR Moderate to vigorous intensity physical activity.ti,ab,kw. OR Energy Expenditure.ti,ab,kw. OR Heart Rate*.ti,ab,kw. OR Pulse Rate*.ti,ab,kw. OR Walking distance.ti,ab,kw. OR Body Composition*.ti,ab,kw. OR Body constitution*.ti,ab,kw. OR Lipid profile*.ti,ab,kw. OR Cholesterol.ti,ab,kw. OR LDL.ti,ab,kw. OR HDL.ti,ab,kw. OR Insulin.ti,ab,kw. OR Glucose.ti,ab,kw. OR Body fat.ti,ab,kw. OR Waist circumference*.ti,ab,kw. OR Anthropometry.ti,ab,kw. OR (Body weights and measures).ti,ab,kw. OR Body Measure*.ti,ab,kw. OR Waist-hip ratio.ti,ab,kw. OR "Metabolic health".ti,ab,kw. OR Health outcome*.ti,ab,kw. OR Body mass index.ti,ab,kw. OR BMI.ti,ab,kw. OR "Health-related quality of life".ti,ab,kw. OR HRQOL.ti,ab,kw. OR exp oxygen consumption/ OR exp aerobic capacity/ OR exp exercise tolerance/ OR exp anthropometric parameters/ OR exp weight reduction/ OR exp blood pressure/ OR exp energy expenditure/ OR exp heart rate and rhythm/ OR exp body composition/ OR exp body constitution/ OR exp cholesterol/ OR exp insulin blood level/ OR exp glucose blood level/ OR exp anthropometry/ OR exp outcome assessment/

**WoS search**

*NCD Population*

"Chronic Disease*" OR "Heart Failure*" OR "Ventricular outflow obstruction*" OR Cardiomyopath* OR "Heart Disease*" OR "Heart Valve Disease*" OR "Myocardial Infarction*" OR "Myocardial Ischemia*" OR "Peripheral Vascular Disease*" OR "Vascular Disease*" OR "Aortic Stenoses" OR "Aortic Stenosis" OR "Aortic Valve Stenos*" OR Atherogenes* OR Atheroscleros* OR Arteriosclerosis OR "Cardiac Disease*" OR "Cardiac rehabilitation*" OR "Cardiovascular disease*" OR "Cardiovascular rehabilitation*" OR "Cardiovascular Stroke" OR CHF OR "Chronic Illness*" OR "Coronary Disease*" OR CVD OR "Heart Attack*" OR "Myocardial Disease*" OR "Myocardial Failure*" OR Myocardiopath* OR "Peripheral Angiopath*" OR "Cardiovascular risk*" OR "Heart dysfunction*" OR "Cardiac dysfunction*" OR Arrhythm* OR "Atrial flutter*" OR Tachycard* OR "Tachyarrhythm*" OR "Arterial disease*" OR "Chronic Airflow Obstruction*" OR "Chronic bronchit*" OR "Lung disease*" OR "Chronic Obstructive Airway Disease*" OR "Chronic Obstructive Lung Disease*" OR "Chronic obstructive pulmonary disease*" OR "Chronic respiratory disease*" OR COAD OR COPD OR Emphysema* OR Diabetes OR "Glucose Intolerance*" OR "Glucose metabolism disorder*" OR "Glucose tolerance*" OR "Glucose Metabolic Disorder*" OR IDDM OR "Impaired fasting glucose" OR Prediabetes OR Pre-diabetes OR "Prediabetic State*" OR Hyperinsulinism* OR Dyslipidemia* OR Dyslipoproteinemia* OR "Dysmetabolic Syndrome X" OR "Elevated Cholesterol*" OR Hypercholesteremia* OR Hypercholesterolemia* OR Hyperlipemia* OR Hyperlipidaemia OR Hyperlipidemia* OR Hypertriglyceridemia* OR Lipemia* OR Lipidemia* OR "Metabolic Cardiovascular Syndrome*" OR "High Cholesterol" OR "High Blood Pressure" OR Hypertension OR Hyperglycemia* OR Hyperinsulinemia OR "Insulin resistance" OR "Insulin sensitivity" OR "Metabolic syndrome*" OR Apoplex* OR "Brain Infarction*" OR "Brain Vascular Accident*" OR "Cerebral Stroke*" OR "Cerebrovascular Accident*" OR CVA OR Stroke* OR "Cerebrovascular disease*" OR "Cerebral Ischemia*" OR "Brain ischemia*" OR "Multiple scleros*" OR "Disseminated Scleros*" OR MS OR "Alzheimer Disease*" OR "Alzheimer's Disease*" OR "Cognition Disorder*" OR "Cognitive Decline*" OR "Cognitive Dysfunction*" OR "Cognitive Impairment*" OR Dement* OR "Neurocognitive Disorder*" OR "Neurological Disorder*"

*Sedentary older adult population*

Septuagenarian* OR Nonagenarian* OR Octogenarian* OR Octagenarian* OR Centenarian* OR Centarian* OR Supercentenarian* OR Elder* OR Eldest OR frail* OR geriatri* OR "old age*" OR "oldest old*" OR senior* OR senium OR "very old*" OR "older people" OR "older subject*" OR "older patient*" OR "older age*" OR "older man" OR "older men" OR "older male*" OR "older woman" OR "older women" OR "older female*" OR "older population*" OR "older person*" OR adult*

*Consumer wearable activity tracker*

TS=(Rehabilitation OR Treatment* OR Intervention* OR Prevention)

**AND**

TS=(Telemedicine OR Telerehabilitation OR "Remote rehabilitation" OR "Mobile Health" OR mHealth OR m-health OR Acceleromet* OR Pedometer*)

TS=("Fitness Tracker*" OR "Activity Tracker*" OR BodyMedia OR DirectLife OR SenseWear OR Fitbit OR Garmin OR Vivosmart OR Jawbone OR "MisFit Shine" OR "Nike FuelBand" OR Polar OR Withings OR Yamax OR Bodybugg OR Tomtom OR Fitbug OR Wahoo OR Omron OR "Apple Watch" OR "Smart wearable*" OR "Activity monitor*" OR "Remote patient monitoring" OR "Fitness device*" OR "Smart watch*" OR "Smart wristband" OR "Smart wearable*" OR Actiwatch)

*Behaviour change*

TS=("Physical Activit*" OR "Health Behavior*" OR "Healthy Behavior*" OR "Health Behaviour*" OR "Healthy Behaviour*" OR "Health Promotion*" OR "Leisure Activit*" OR "Physical Exercis*" OR "Measure activit*" OR "Behavior Modification*" OR "Behaviour Modification*" OR "Behavioral Modification*" OR "Behavioural Modification*" OR "Behavior change*" OR "Behaviour change*" OR "Motor Activit*" OR Sedentary OR "Leisure time" OR treatment* OR "lifestyle modification*" OR "lifestyle change*")

*Cardiometabolic health outcomes*

TS=("Physical fitness" OR "Exercise tolerance" OR "Blood pressure" OR "Heart rate*" OR "Body composition*" OR "Body constitution*" OR "Cholesterol" OR "Body fat" OR "Waist circumference*" OR "Body weights and measures" OR "Waist-hip ratio*" OR "Waist-to-hip ratio*" OR Anthropometry OR "Body Mass Index" OR "Oxygen uptake" OR VO2 OR Weight OR "Diastolic Pressure" OR "Systolic Pressure" OR "Pulse Pressure" OR Steps OR "Step count" OR MVPA OR "Moderate to vigorous intensity*" OR "Energy Expenditure" OR "Pulse Rate*" OR "Walking distance*" OR "Lipid profile*" OR HDL OR LDL OR "insulin" OR "glucose" OR "Body Measure*" OR "Metabolic health" OR "Health outcome*" OR "Body mass index" OR BMI)

**CINAHL search**

*NCD Population*

MH "Cardiovascular Diseases+" OR MH "Chronic Disease" OR "Chronic Disease*" OR "Heart Failure*" OR "Ventricular outflow obstruction*" OR Cardiomyopath* OR "Heart Disease*" OR "Heart Valve Disease*" OR "Myocardial Infarction*" OR "Myocardial Ischemia*" OR "Peripheral Vascular Disease*" OR "Vascular Disease*" OR "Aortic Stenoses" OR "Aortic Stenosis" OR "Aortic Valve Stenos*" OR Atherogenes* OR Atheroscleros* OR Arteriosclerosis OR "Cardiac Disease*" OR "Cardiac rehabilitation*" OR "Cardiovascular disease*" OR "Cardiovascular rehabilitation*" OR "Cardiovascular Stroke" OR CHF OR "Chronic Illness*" OR "Coronary Disease*" OR CVD OR "Heart Attack*" OR "Myocardial Disease*" OR "Myocardial Failure*" OR Myocardiopath* OR "Peripheral Angiopath*" OR "Cardiovascular risk*" OR "Heart dysfunction*" OR "Cardiac dysfunction*" OR Arrhythm* OR "Atrial flutter*" OR Tachycard* OR "Tachyarrhythm*" OR "Arterial disease*" OR MH "Lung diseases+" OR "Chronic Airflow Obstruction*" OR "Chronic bronchit*" OR "Lung disease*" OR "Chronic Obstructive Airway Disease*" OR "Chronic Obstructive Lung Disease*" OR "Chronic obstructive pulmonary disease*" OR "Chronic respiratory disease*" OR COAD OR COPD OR Emphysema* OR MH "Glucose Metabolism Disorders+" OR MH "Insulin resistance+" OR Diabetes OR "Glucose Intolerance*" OR "Glucose metabolism disorder*" OR "Glucose tolerance*" OR "Glucose Metabolic Disorder*" OR IDDM OR "Impaired fasting glucose" OR Prediabetes OR Pre-diabetes OR "Prediabetic State*" OR MH "hyperlipidemia+" OR Hyperinsulinism* OR Dyslipidemia* OR Dyslipoproteinemia* OR "Dysmetabolic Syndrome X" OR "Elevated Cholesterol*" OR Hypercholesteremia* OR Hypercholesterolemia* OR Hyperlipemia* OR Hyperlipidaemia OR Hyperlipidemia* OR Hypertriglyceridemia* OR Lipemia* OR Lipidemia* OR "Metabolic Cardiovascular Syndrome*" OR "High Cholesterol" OR "High Blood Pressure" OR Hypertension OR Hyperglycemia* OR Hyperinsulinemia OR "Insulin resistance" OR "Insulin sensitivity" OR "Metabolic syndrome*" OR MH "Obesity+" OR Obes* OR Overweight* OR MH "Cerebrovascular Disorders+" OR Apoplex* OR "Brain Infarction*" OR "Brain Vascular Accident*" OR "Cerebral Stroke*" OR "Cerebrovascular Accident*" OR CVA OR Stroke* OR "Cerebrovascular disease*" OR "Cerebral Ischemia*" OR "Brain ischemia*" OR MH "Multiple sclerosis" OR "Multiple scleros*" OR "Disseminated Scleros*" OR MS OR "Alzheimer Disease*" OR "Alzheimer's Disease*" OR "Cognition Disorder*" OR "Cognitive Decline*" OR "Cognitive Dysfunction*" OR "Cognitive Impairment*" OR Dement* OR "Neurocognitive Disorder*" OR "Neurological Disorder*"

*Sedentary older adult population*

MH "Postmenopause" OR MH "Aged+" OR Septuagenarian* OR Nonagenarian* OR Octogenarian* OR Octagenarian* OR Centenarian* OR Centarian* OR Supercentenarian* OR Elder* OR Eldest OR frail* OR geriatri* OR "old age*" OR "oldest old*" OR senior* OR senium OR "very old*" OR "older people" OR "older subject*" OR "older patient*" OR "older age*" OR "older man" OR "older men" OR "older male*" OR "older woman" OR "older women" OR "older female*" OR "older population*" OR "older person*" OR adult*

*Consumer wearable activity tracker*

Rehabilitation OR Treatment* OR Intervention* OR Prevention

**AND**

MH "Telemedicine+" OR MH "Wearable electronic devices+" OR Telemedicine OR Telerehabilitation OR "Mobile Health" OR mHealth OR m-health OR Acceleromet* OR Pedometer*

"Fitness Tracker*" OR "Activity Tracker*" OR "Smart wearable*" OR "Activity monitor*" OR “Remote patient monitoring” OR "Fitness device*" OR BodyMedia OR DirectLife OR Sensewear OR Fitbit OR Garmin OR Vivosmart OR Jawbone OR "MisFit Shine" OR "Nike FuelBand" OR Polar OR Withings OR Yamax OR Bodybugg OR Tomtom OR Fitbug OR Wahoo OR Omron OR "Apple Watch" OR "Smart watch*" OR "Smart wristband" OR Actiwatch

*Behaviour change*

MH "Health promotion" OR MH "Leisure activities+" OR MH "Motor activity" OR "Physical Activit*" OR "Health Behavior*" OR "Healthy Behavior*" OR "Health Behaviour*" OR "Healthy Behaviour*" OR "Health Promotion*" OR "Leisure Activit*" OR "Physical Exercis*" OR "Measure activit*" OR "Behavior Modification*" OR "Behaviour Modification*" OR "Behavioral Modification*" OR "Behavioural Modification*" OR "Behavior change*" OR "Behaviour change*" OR "Motor Activit*" OR Sedentary OR "Leisure time" OR treatment* OR "lifestyle modification*" OR "lifestyle change*"

*Cardiometabolic health outcomes*

MH "cardiorespiratory fitness" OR MH "Exercise tolerance+" OR MH "Blood pressure+" OR MH "Heart rate" OR MH "Body weights and measures+" OR MH "Body constitution+" OR MH "Cholesterol+" OR MH "Anthropometry" OR "Physical fitness" OR "Exercise tolerance" OR "Blood pressure" OR "Heart rate*" OR "Body composition*" OR "Body constitution*" OR "Cholesterol" OR "Body fat" OR "Waist circumference*" OR "Body weights and measures" OR "Waist-hip ratio*" OR "Waist-to-hip ratio*" OR Anthropometry OR "Body Mass Index" OR "Oxygen uptake" OR VO2 OR Weight OR "Diastolic Pressure" OR "Systolic Pressure" OR "Pulse Pressure" OR Steps OR "Step count" OR MVPA OR "Moderate to vigorous intensity*" OR "Energy Expenditure" OR "Pulse Rate*" OR "Walking distance*" OR "Lipid profile*" OR HDL OR LDL OR "insulin" OR "glucose" OR "Body Measure*" OR "Metabolic health" OR "Health outcome*" OR "Body mass index" OR BMI

**CENTRAL search**

*NCD Population*

"Cardiac Rehabilitation"[MeSH] OR "Cardiovascular Diseases"[MeSH] OR "Chronic Disease"[MeSH] OR "Aortic Stenoses" OR "Aortic valve disease*" OR "Aortic Valve Stenos*" OR Arteriosclerosis OR Atherogenesis OR Atheroscleroses OR Atherosclerosis OR "Cardiac Disease*" OR "Cardiac dysfunction*" OR "Cardiac rehabilitation*" OR Cardiomyopath* OR "Cardiovascular disease*" OR "Cardiovascular rehabilitation*" OR "Cardiovascular risk*" OR "Cardiovascular Stroke" OR CHF OR "Chronic disease*" OR "Chronic Illness*" OR "Coronary Disease*" OR CVD OR "Heart Attack*" OR "Heart Disease*" OR "Heart dysfunction*" OR "Heart Failure*" OR "Heart Valve Disease*" OR "Myocardial Disease*" OR "Myocardial Failure*" OR "Myocardial Infarction*" OR "Myocardial Ischemia*" OR Myocardiopath* OR "Peripheral Angiopath*" OR "Vascular Disease*" OR "Ventricular outflow obstruction*" OR Arrhythm* OR "Atrial flutter*" OR Tachycard* OR Tachyarrhythm* OR "Arterial disease*" OR "Lung diseases"[MeSH] OR "Chronic Airflow Obstruction*" OR "Chronic bronchitis" OR "Chronic Obstructive Airway Disease*" OR "Chronic obstructive pulmonary disease*" OR "Chronic respiratory disease*" OR COAD OR COPD OR Emphysema* OR "Lung disease*" OR "Glucose Metabolism Disorders"[MeSH] OR Diabetes OR "Glucose Intolerance*" OR "Glucose metabolism disorder*" OR "Glucose tolerance*" OR "Glucose Metabolic Disorder*" OR IDDM OR "Impaired fasting glucose" OR Prediabetes OR Pre-diabetes OR "Prediabetic State*" OR "Dyslipidemias"[MeSH] OR Dyslipidemia* OR Dyslipoproteinemia* OR "Dysmetabolic Syndrome X" OR "Elevated Cholesterol*" OR "High Cholesterol Level*" OR Hypercholesteremia* OR Hypercholesterolemia* OR Hyperlipemia* OR Hyperlipidaemia OR Hyperlipidemia* OR Hypertriglyceridemia* OR Lipemia* OR Lipidemia* OR "Metabolic Cardiovascular Syndrome*" OR "High Cholesterol" OR "High Blood Pressure" OR Hypertension OR Hyperglycemia* OR Hyperinsulinemia OR "Insulin resistance" OR "Insulin sensitivity" OR "Metabolic syndrome*" OR "Overweight"[MeSH] OR Obes* OR Overweight* OR "Cerebrovascular Disorders"[MeSH] OR Apoplex* OR "Brain Infarction*" OR "Brain Vascular Accident*" OR "Cerebrovascular Accident*" OR CVA OR Stroke* OR "Cerebrovascular disease*" OR "Cerebral Ischemia*" OR "Brain ischemia*" OR "Multiple sclerosis"[MeSH] OR "Disseminated Scleros*" OR MS OR "Multiple Scleros*" OR "Neurocognitive disorders"[MeSH] OR "Alzheimer Disease*" OR "Alzheimer's Disease*" OR "Cognition Disorder*" OR "Cognitive Decline*" OR "Cognitive Dysfunction*" OR "Cognitive Impairment*" OR "Cognitive defect*" OR Dement* OR "Neurocognitive Disorder*" OR "Neurological Disorder*"

*Sedentary older adult population*

"Postmenopause"[MeSH] OR "Adult"[MeSH] OR Septuagenarian* OR Nonagenarian* OR Octogenarian* OR Octagenarian* OR Centenarian* OR Centarian* OR Supercentenarian* OR Elder* OR frail* OR geriatri* OR "old age*" OR "oldest old*" OR senior* OR senium OR "very old*" OR "older people" OR "older subject*" OR "older patient*" OR "older age*" OR "older adult*" OR "older man" OR "older men" OR "older male*" OR "older woman" OR "older women" OR "older female*" OR "older population*" OR "older person*" OR adult* OR postmenopaus*

*Consumer wearable activity tracker*

Rehabilitation OR Treatment* OR Intervention* OR Prevention

**AND**

"Telemedicine"[MeSH] OR "Telerehabilitation"[MeSH] OR "Wearable electronic devices"[MeSH] OR Telemedicine OR Telerehabilitation OR "Remote Rehabilitation" OR "Mobile Health" OR mHealth OR m-health OR Pedometer OR Acceleromet*

**OR**

"Fitness Tracker*" OR "Activity Tracker*" OR "Smart wearable*" OR "Activity monitor*" OR "Remote patient monitoring" OR "Fitness device*" OR SenseWear OR "BodyMedia Fit" OR DirectLife OR Fitbit OR Garmin OR Vivosmart OR Jawbone OR "MisFit Shine" OR "Nike FuelBand" OR Polar OR Withings OR Yamax OR Bodybugg OR Tomtom OR Fitbug OR Wahoo OR Omron OR "Apple Watch" OR "Smart watch*" OR "Smart wristband" OR "actiwatch"

*Behaviour change*

"Health behavior"[MeSH] OR "Health promotion"[MeSH] OR "Leisure activities"[MeSH] OR "Motor activity"[MeSH] OR "Physical Activit*" OR "Health Behavior*" OR "Healthy Behavior*" OR "Health Behaviour*" OR "Healthy Behaviour*" OR "Health Promotion*" OR "Leisure Activit*" OR "Physical Exercis*" OR "Measure activit*" OR "Behavior modification*" OR "Behaviour modification*" OR "Behavioral modification*" OR "behavioural modification*" OR "Behavior change*" OR "Behaviour change*" OR "Motor Activit*" OR Sedentary OR "Leisure time" OR "lifestyle modification*" OR "lifestyle change*"

*Cardiometabolic health outcomes*

"Physical fitness"[MeSH] OR "Exercise tolerance"[MeSH] OR "Blood pressure"[MeSH] OR "Heart rate"[MeSH] OR "Body weights and measures"[MeSH] OR "Body constitution"[MeSH] OR "Cholesterol"[MeSH] OR "Fat body"[MeSH] OR "Anthropometry"[MeSH] OR "Oxygen uptake" OR "Oxygen consumption" OR VO2 OR "Physical Fitness" OR "Exercise tolerance" OR Weight OR "Blood Pressure" OR "Diastolic Pressure" OR "Systolic Pressure" OR "Pulse Pressure" OR Steps OR "Step count" OR MVPA OR "Moderate to vigorous intensity*" OR "Energy Expenditure" OR "Heart Rate*" OR "Pulse Rate*" OR "Walking distance" OR "Body Composition*" OR "Body Constitution*" OR "Lipid profile*" OR Cholesterol OR LDL OR HDL OR Insulin OR Glucose OR "Body fat" OR "Waist Circumference*" OR "Body Measure*" OR "Waist-Hip Ratio*" OR "Waist-to-hip ratio*" OR Anthropometr* OR "Metabolic health" OR "Health outcome*" OR BMI OR "Body mass index"
