# Supplementary material for: Can consumer wearable activity tracker-based interventions improve physical activity and cardiometabolic health in patients with chronic diseases? A systematic review and meta-analysis of randomised controlled trials
Source: Int J Behav Nutr Phys Act. 2020 May 11;17:57. doi: 10.1186/s12966-020-00955-2 (PMC7216601; doi:10.1186/s12966-020-00955-2)
Supplement: Supplementary file 2 — Additional file 2: Appendix II Data extraction form. [file 12966_2020_955_MOESM2_ESM.docx]

**Data Extraction Form adapted from the Cochrane Collaboration**

**Title of the systematic review:** Can consumer wearable activity trackers improve physical activity and cardiometabolic health in patients with chronic diseases?: A systematic review and meta-analysis of randomized controlled trials

**Version number:** 20180424

| **1. Data form completed**  **(dd/mm/yyyy)** |  |
| --- | --- |
| **2. Name/ID of person extracting data** |  |
| **3. Report title (title of paper/abstract/ report that data are extracted from)** |  |
| **4. Report contact details of person extracting data** |  |
| **5. Publication type (e.g. full report, abstract, letter)** |  |
| **6. Study ID (e.g. Surname of first author and year first full report of study was published e.g. Smith 2001)** |  |
| **7. Country in which the study was performed** |  |
| **8. Economic level of the country in which the study was performed (e.g. low income, lower-middle income or upper-middle income)** |  |
| **9. Study funding source** |  |
| **10. Possible conflicts of interest** |  |
| **Notes:** | |

| **Study Characteristics** | **Review Inclusion Criteria** | **Location in text (page#/fig/table)** |
| --- | --- | --- |
| **11. Type of study** |  |  |
| **12. Population description** |  |  |
| **13. Focused disease/condition** |  |  |
| **14. Types of outcomes measures**  **(Prevalence/Risk factors)** |  |  |
| **15. Notes:** | | |

| **Study Participants** | **Description** | **Location in text (page#/fig/table)** |
| --- | --- | --- |
| **16. Total sample size** |  |  |
| **17. Age** |  |  |
| **18. Sex** |  |  |
| **19. Country** |  |  |
| **20. Source/setting of the population (e.g. urban, rural)** |  |  |
| **21. Ethnicity** |  |  |
| **22. Notes:** | | |

| **Methods** | **Description** | **Location in text (page#/fig/table)** |
| --- | --- | --- |
| **23. Aim of the study** |  |  |
| **24. Study design (e.g. cross-sectional study, case-control study)** |  |  |
| **25. Duration of intervention** |  |  |
| **26. Intervention type** |  |  |
| **27. Intervention frequency** |  |  |
| **28. CWAT type** |  |  |
| **26. Blinding** |  |  |
| **27. Notes:** | | |

| **Interventions** | **Description** | **Location in text (page#/fig/table)** |
| --- | --- | --- |
| **28. Total number of intervention groups** |  |  |
| **29. Notes:** | | |

| **Intervention 1** | **Description** | **Location in text (page#/fig/table)** |
| --- | --- | --- |
| **30. Specific intervention** |  |  |
| **31. Notes:** | | |

| **Outcomes** | **Description** | **Location in text (page#/fig/table)** |
| --- | --- | --- |
| **36. physical examination/ Self-reported outcomes** |  |  |
| **37. Notes:** | | |

| **Outcome 1** | **Description** | **Location in text (page#/fig/table)** |
| --- | --- | --- |
| **38. Outcome definition** |  |  |
| **39. Unit of measurement** |  |  |
| **40. Time points measured** |  |  |
| **41. Time points reported** |  |  |
| **42. Statistical method used** |  |  |
| **43. Notes:** | | |

| **Outcome 2** | **Description** | **Location in text (page#/fig/table)** |
| --- | --- | --- |
| **44. Outcome definition** |  |  |
| **45. Unit of measurement** |  |  |
| **46. Time points measured** |  |  |
| **47. Time points reported** |  |  |
| **48. Statistical method used** |  |  |
| **49. Notes:** | | |

| **Outcome 3** | **Description** | **Location in text (page#/fig/table)** |
| --- | --- | --- |
| **50. Outcome definition** |  |  |
| **51. Unit of measurement** |  |  |
| **52. Time points measured** |  |  |
| **53. Time points reported** |  |  |
| **54. Statistical method used** |  |  |
| **55. Notes:** | | |

| **Results** | **Description** | **Location in text (page#/fig/table)** |
| --- | --- | --- |
| **56. Number of participants allocated to each intervention group** |  |  |
| **57. Notes:** | | |

| **Outcome 1** | **Description as stated in paper** | **Location in text (page#/fig/table)** |
| --- | --- | --- |
| **58. Outcome name** |  |  |
| **59. Sample size** |  |  |
| **60. Missing participants** |  |  |
| **61. Summary data for intervention group (e.g. mean and SDs)** |  |  |
| **62. Estimate of effect with confidence interval; P value** |  |  |
| **63. Notes:** | | |

| **Outcome 2** | **Description as stated in paper** | **Location in text (page#/fig/table)** |
| --- | --- | --- |
| **64. Outcome name** |  |  |
| **65. Sample size** |  |  |
| **66. Missing participants** |  |  |
| **67. Summary data for intervention group (e.g. mean and SDs)** |  |  |
| **68. Estimate of effect with confidence interval; P value** |  |  |
| **69. Notes:** | | |

| **Outcome 3** | **Description as stated in paper** | **Location in text (page#/fig/table)** |
| --- | --- | --- |
| **70. Outcome name** |  |  |
| **71. Sample size** |  |  |
| **72. Missing participants** |  |  |
| **73. Summary data for intervention group (e.g. mean and SDs)** |  |  |
| **74. Estimate of effect with confidence interval; P value** |  |  |
| **75. Notes:** | | |

| **Limitations** | **Description as stated in paper** | **Location in text (page#/fig/table)** |
| --- | --- | --- |
| **76. Strength** |  |  |
| **77. Limitations** |  |  |
| **78. Notes:** | | |

| **Conclusion** | **Description as stated in paper** | **Location in text (page#/fig/table)** |
| --- | --- | --- |
| **79. Key conclusions of the study authors** |  |  |
| **80. Notes:** | | |
