# Supplementary material for: Can consumer wearable activity tracker-based interventions improve physical activity and cardiometabolic health in patients with chronic diseases? A systematic review and meta-analysis of randomised controlled trials
Source: Int J Behav Nutr Phys Act. 2020 May 11;17:57. doi: 10.1186/s12966-020-00955-2 (PMC7216601; doi:10.1186/s12966-020-00955-2)
Supplement: Supplementary file 3 — Additional file 3: Appendix III Funnel plots. [file 12966_2020_955_MOESM3_ESM.pdf]

Appendix III Funnel plots

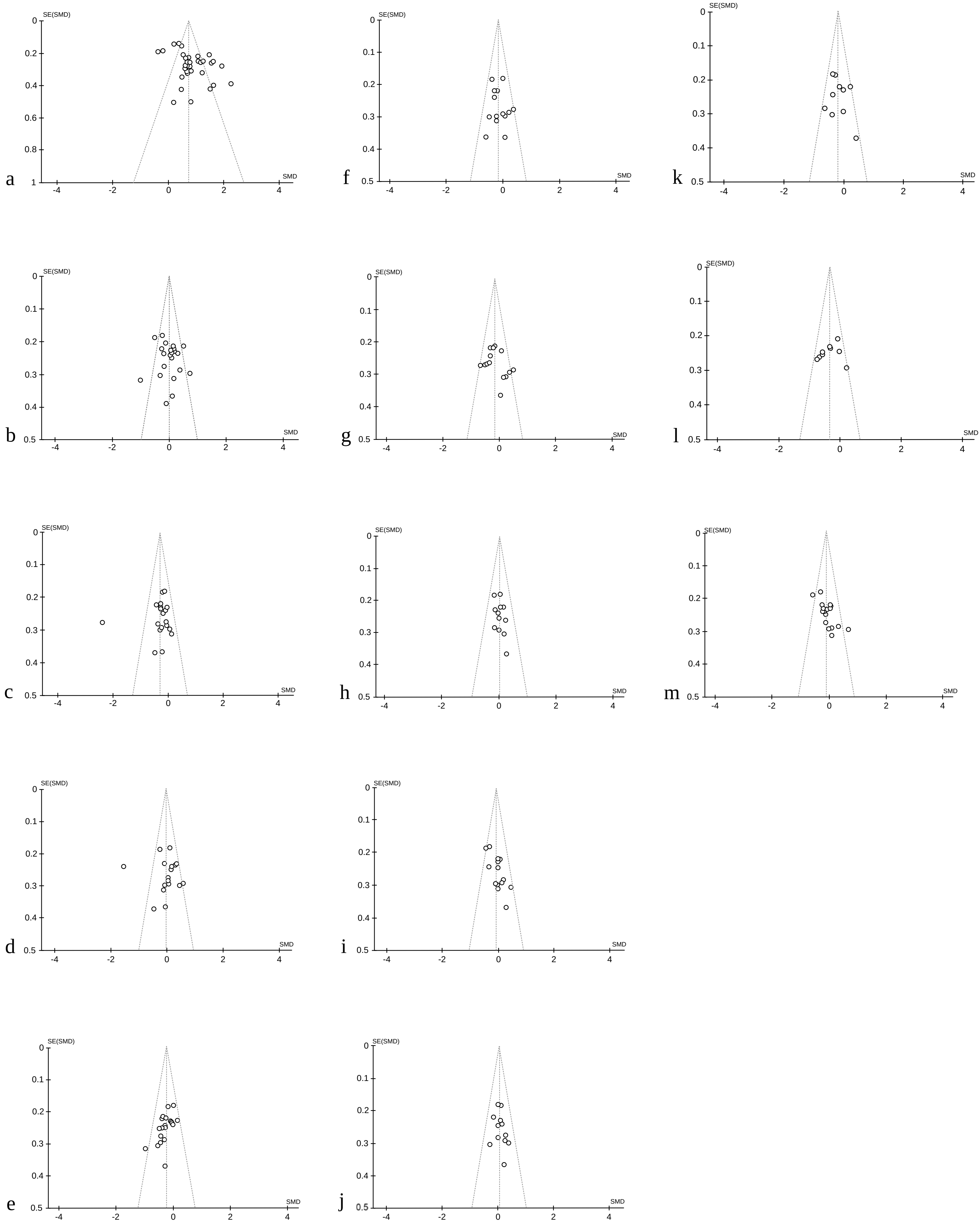

Funnel plots comparing interventions with regard to (a) physical activity, (b) body mass index, (c) systolic blood pressure, (d) diastolic blood pressure, (e) waist circumference, (f) HbA1c, (g) fasting glucose concentration, (h) triglyceride concentration, (i) total cholesterol concentration, (j) high-density lipoprotein cholesterol concentration, (k) low-density cholesterol concentration, (l) resting heart rate and (m) body weight.
